# Proteome and Membrane Fatty Acid Analyses on *Oligotropha carboxidovorans* OM5 Grown under Chemolithoautotrophic and Heterotrophic Conditions

**DOI:** 10.1371/journal.pone.0017111

**Published:** 2011-02-28

**Authors:** Debarati Paul, Ranjit Kumar, Bindu Nanduri, Todd French, Ken Pendarvis, Ashli Brown, Mark L. Lawrence, Shane C. Burgess

**Affiliations:** 1 College of Veterinary Medicine, Mississippi State University, Mississippi State, Mississippi, United States of America; 2 Department of Chemical Engineering, Mississippi State University, Mississippi State, Mississippi, United States of America; 3 Life Sciences and Biotechnology Institute, Mississippi State University, Mississippi State, Mississippi, United States of America; 4 Department of Biochemistry and Molecular Biology, Mississippi State University, Mississippi State, Mississippi, United States of America; University of Kansas, United States of America

## Abstract

*Oligotropha carboxidovorans* OM5 T. (DSM 1227, ATCC 49405) is a chemolithoautotrophic bacterium able to utilize CO and H_2_ to derive energy for fixation of CO_2_. Thus, it is capable of growth using syngas, which is a mixture of varying amounts of CO and H_2_ generated by organic waste gasification. *O. carboxidovorans* is capable also of heterotrophic growth in standard bacteriologic media. Here we characterize how the *O. carboxidovorans* proteome adapts to different lifestyles of chemolithoautotrophy and heterotrophy. Fatty acid methyl ester (FAME) analysis of *O. carboxidovorans* grown with acetate or with syngas showed that the bacterium changes membrane fatty acid composition. Quantitative shotgun proteomic analysis of *O. carboxidovorans* grown in the presence of acetate and syngas showed production of proteins encoded on the megaplasmid for assimilating CO and H_2_ as well as proteins encoded on the chromosome that might have contributed to fatty acid and acetate metabolism. We found that adaptation to chemolithoautotrophic growth involved adaptations in cell envelope, oxidative homeostasis, and metabolic pathways such as glyoxylate shunt and amino acid/cofactor biosynthetic enzymes.

## Introduction

*Oligotropha carboxidovorans* OM5, the only species representing the genus *Oligotropha*, is capable of growth under chemolithoautotrophic conditions by oxidizing CO+H_2_, and it is capable also of growing heterotrophically [1], [2], [3]. Strain OM5 harbors the low-copy-number 133 kb megaplasmid pHCG3 that carries four gene clusters: *cox* (14.54 kb), *hox* (23.35 kb), *cbb* (13.33 kb), and *tra/trb* (25.74 kb) [1]. These gene clusters encode the functions required for chemolithoautotrophic utilization of CO and H_2_, CO_2_ fixation, and conjugal transfer of the plasmid, respectively.

Our completed OM5 chromosome sequence [3] shows that this bacterium encodes putative metabolic enzymes involved in energy metabolism, biosynthesis of amino acids/fatty acids, nucleotide metabolism, transcription, protein synthesis and degradation, and signal transduction. The chromosome also contains genes encoding putative enzymes that allow heterotrophic growth with organic acids, such as acetate, pyruvate, lactate, crotonate, malate, succinate, formate, and glyoxylate as substrates [3].

*O. carboxidovorans* metabolism can vary considerably because it can grow under chemolithoautotrophic or heterotrophic conditions [1], [3]. During growth under chemolithoautotrophic conditions, it utilizes the reducing power of CO and H_2_ to fix carbon using ribulose bisphosphate carboxylase (RuBisCo) [4]. During heterotrophic growth, it utilizes fixed carbon compounds as carbon and energy source, and its metabolism can vary based on the carbon source. When sugars are available, they feed directly into glycolysis and the TCA cycle. In some bacteria, including strain OM5, acetate from the medium can be used as a sole energy source by converting it to sugars and fatty acids via the glyoxylate cycle [5], [6].

The ability of *O. carboxidovorans* to utilize CO and H_2_ makes it capable of growing in syngas (synthesis gas [7]), a gas mixture that contains varying amounts of carbon monoxide, some carbon dioxide, and hydrogen. Though syngas is combustible and often used as a fuel source, it often has less than half the energy density of natural gas. *O. carboxidovorans* is of interest for bioenergy production because potentially it can utilize syngas produced from cellulose and hemicellulose wastes (biomass) to produce a biofuel substrate.

Here we demonstrate that *O. carboxidovorans* adapts to growth under heterotrophic and chemolithoautotrophic conditions by managing its proteome and fatty acid composition. We characterized the *O. carboxidovorans* proteome using quantitative shotgun proteomics under three contrasting conditions: growth on a complex growth medium, growth in a minimal medium with only acetate as a fixed carbon source, and growth in minimal medium with syngas. We also conducted fatty acid methyl ester (FAME) analysis of *O. carboxidovorans* under the same conditions. Our results increase our understanding of *O. carboxidovorans* metabolism during heterotrophic and autotrophic growth and in particular provide information on proteomic changes during utilization of syngas for bioenergy.

## Results and Discussion

### Bacterial growth

**Figure 1:**
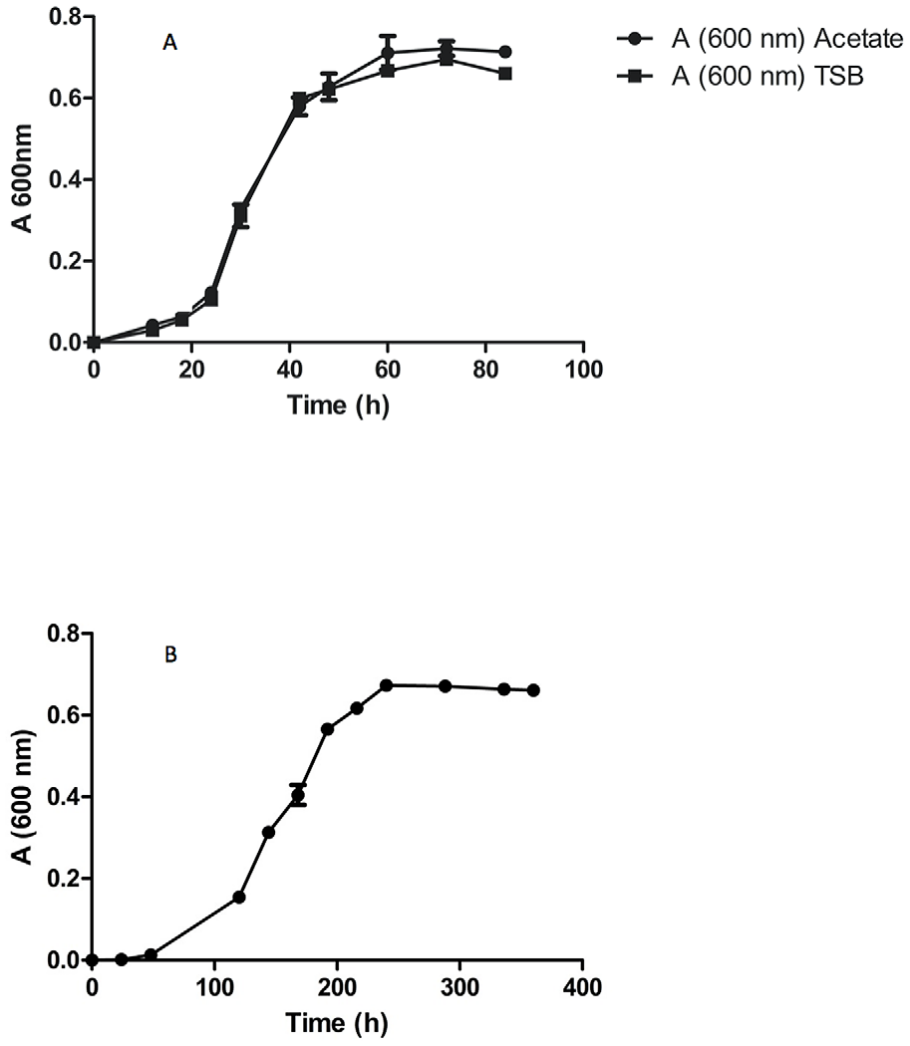
Growth curves of strain OM5 under the three different growth conditions. (A) acetate medium and TSB; (B) syngas. Error bars indicate standard error.

As expected, *O. carboxidovorans* showed different growth patterns depending on growth conditions. While growing in nutrient rich TSB, the bacteria attained an OD_600_ of 0.6 in ∼48 h. In minimal medium containing acetate, the time required was ∼44 h. *O. carboxidovorans* has enzymes for the glyoxylate shunt for acetate utilization, but growth in acetate medium was surprisingly efficient. By contrast, growth in minimal medium in the presence of syngas was much slower, requiring ∼240 h (Figure 1). This result agrees with earlier findings [4]. Growth in syngas may prolong lag phase to allow the bacteria to produce enzymes required for oxidizing CO and H_2_ as well as fixing CO_2_. Doubling time in log phase is probably also increased in syngas because of the needs to derive energy from oxidization of inorganic molecules and to fix carbon.

### FAME analysis

The nutritional differences produced differences in *O. carboxidovorans* fatty acid profile. *O. carboxidovorans* respond to growth in syngas with homeoviscous adaptation (a homeostatic process that regulates the viscosity of membrane lipids): the fatty acid profile shifted toward long chain fatty acids. FAME analysis (Table 1) showed that the amount of octadecanoic acid and octadecenoic acid increased in syngas and acetate relative to TSB, and these were the most abundant fatty acids present when grown in acetate. In syngas, hexadecanoic acid and hexadecenoic acid were the most abundant. In TSB, hexadecenoic acid was absent. Instead, octanoic acid and octadecatrienoic acid were present (these were not identified during acetate and syngas growth).

**Table 1:** Results of FAME analysis.

Fatty acid methyl ester	Common name	Amount (ng/ml)		
		TSB	SYNGAS	ACETATE
Octanoic acid methyl ester	Caprylic acid	6.337	0	0
Hexadecanoic acid methyl ester	**Palmitic acid** *	30.05	105.3	19.91
Hexadecenoic acid methyl ester	Palmitoleic acid	0	55.16	18.99
Octadecanoic acid methyl ester	**Stearic acid**	10.5	14.37	33.89
Octadecenoic acid methyl ester	**Oleic acid**	12.58	40.77	58.20
Octadecatrienoic acid methyl ester	Α-Linolenic acid	1.373	0	0

*Fatty acids in bold are biodiesel components.

Unlike oleaginous organisms (which normally contain 20–25% oil), *O. carboxidovorans* cannot store fatty acids. The presence of long chain fatty acids in the membrane might be due to the slower turnover of fats or an active response to changes in environmental conditions and/or growth substrates for the cell. Different carbon sources affect bacterial fatty acid composition and, in turn, cell membrane permeability and fluidity [8], [9], [10]. Long chain fatty acids and unbranched saturated fatty acids pack more tightly and decrease membrane fluidity [8], [10]. There is precedent for bacteria altering membrane fatty acid composition in response to changes in carbon source: *Acenitobacter* HHI-1 increases fatty acid 16:1 content based on carbon source [8]. However, this is not a universal trend; *E.* coli lipid and fatty acid composition is independent of carbon source (glucose, glycerol, succinate, acetate, fatty acids and amino acids) [11]. In Gram-negative bacteria, stress can also change the ratio of saturated to unsaturated fatty acids, cause an increase in the ratio of trans- to cis-monoenoic fatty acids, and increase the ratio of cyclopropyl to monoenoic precursor fatty acids [12]. One caveat is that because TSB and acetate cultures were grown in flasks with rotary shaking, shear forces may have been responsible for some of the effects on fatty acid composition that we detected; however, we have found no published data to support this supposition.

### Proteome coverage

**Figure 2:**
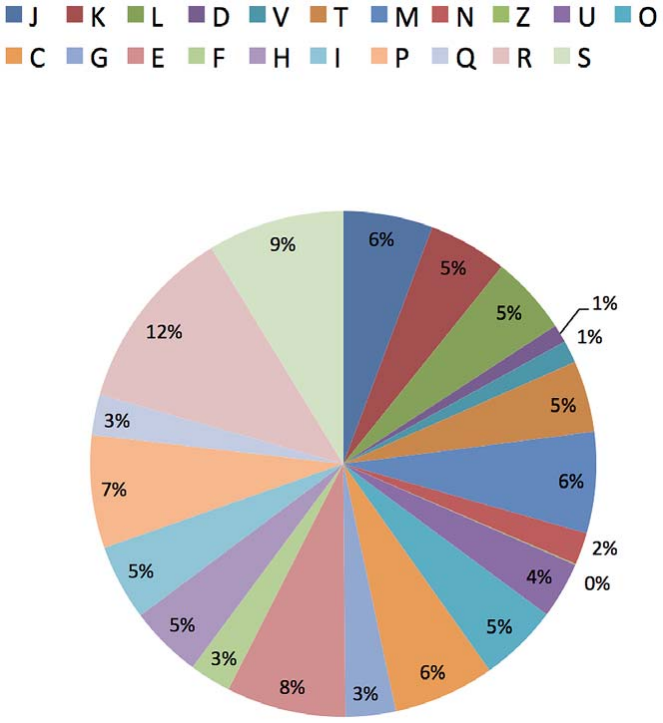
Pie chart showing the distribution of identified proteins (combined from all three growth conditions) in COG categories. J Translation; K Transcription; L Replication, recombination and repair; D Cell cycle control; T Signal transduction mechanism; M Cell wall/membrane biogenesis; N Cell motility; Z Cytoskeleton; U Intracellular trafficking and secretion; O Posttranslational modification, protein turnover, chaperones; C Energy production and conversion; G Carbohydrate transport and metabolism; E Carbohydrate transport and metabolism; F Nucleotide transport and metabolism; H Coenzyme transport and metabolism; I Lipid transport and metabolism; Q Secondary metabolites biosynthesis, transport and catabolism; R General function prediction only; S Function unknown. The COG categories were unknown for plasmid encoded proteins and 518 chromosomal proteins.

**Figure 3:**
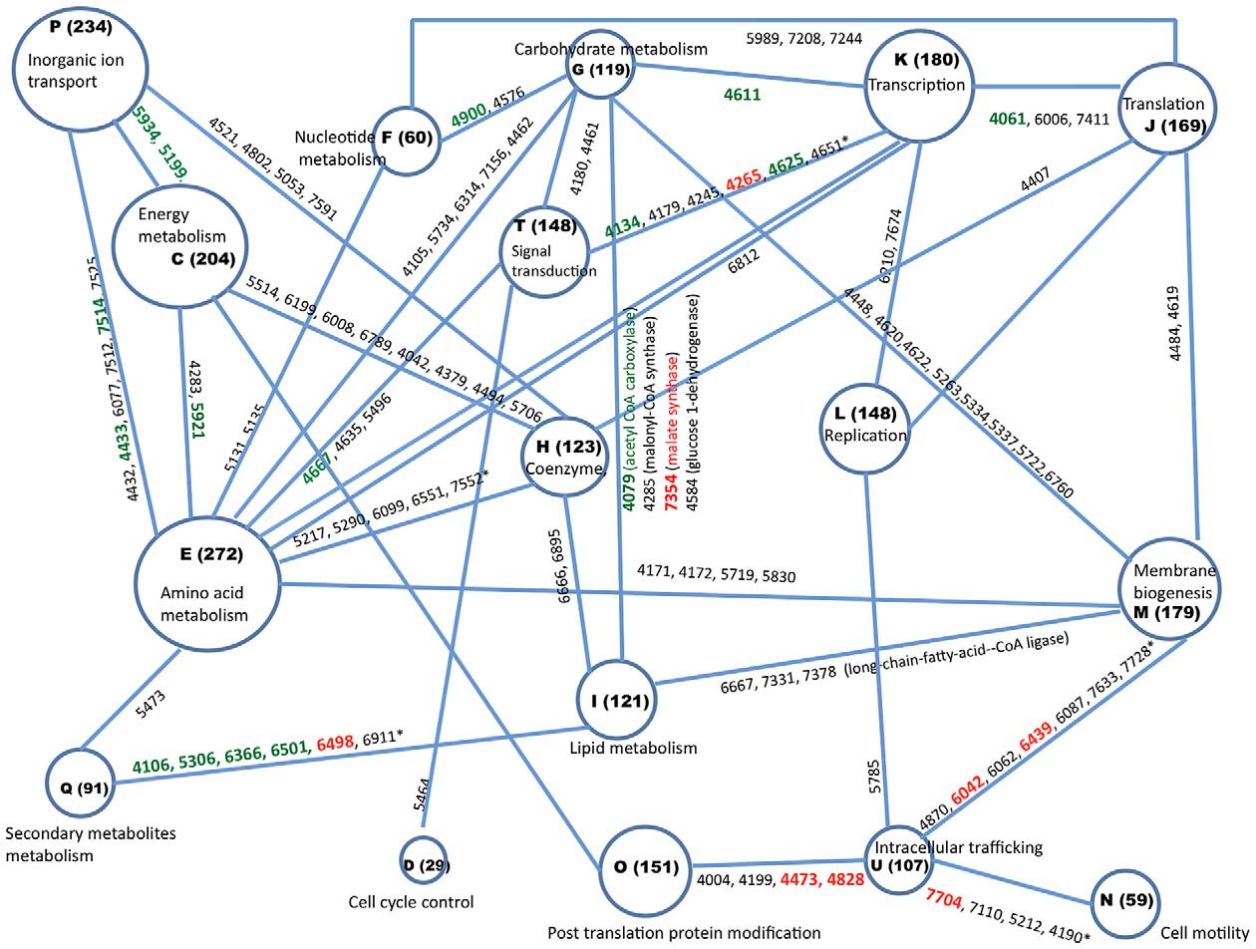
Metabolic network showing the number of *O. carboxidovorans* proteins identified in each COG category in the current study. Proteins (referred by locus tag number OCAR_xxxx) listed on lines connecting COG categories are classified in both COG categories. Proteins that showed significantly increased expression in syngas and acetate medium compared to TSB medium are colored red, and proteins that had significantly decreased expression in syngas and actetate medium are green. The size of the circles represents the number of proteins in that COG. Protein names and COG groups along with their GI and other details can be accessed from NCBI (http://www.ncbi.nlm.nih.gov/genome?Db=genome&Cmd=Retrieve&dopt=ProteinTable&list_uids=23052). The COG categories indicated are the same as in Figure 2. *Indicates additional proteins classified in both COG categories not shown in the figure.

In all, 1317 proteins were identified in TSB-grown bacteria, 1091 in acetate-grown, and 634 in syngas-grown. Although *O. carboxidovorans* metabolism is likely to be quite different when it grows in nutrient rich TSB medium versus growth in minimal medium with acetate as a sole carbon source, there were 668 proteins expressed during both of these heterotrophic growth conditions. By contrast, there were many fewer proteins shared between growth in syngas and the other two growth conditions. Of the total 1977 proteins identified, only 190 were present in all three growth conditions, demonstrating the greater degree of change in the proteome required when *O. carboxidovorans* adapts from heterotrophy to chemolithoautotrophy. Figure 2 shows the distribution of identified proteins in various COG categories. Figure 3 shows a metabolic network of various proteins that interconnect these COG categories and also highlights a few that were important to this study.

Heatmaps (Figure S1) show, as one would expect, that each growth condition has a uniquely-expressed proteome and that there are areas of shared protein expression and regulation. Furthermore, these areas can be clustered by function. Complete lists of all the proteins with significantly altered quantities under the three growth conditions are available in Tables S1, S2, S3, S4, S5, and S6.

### Growth phase proteins

Although bacteria were harvested at the same stage of growth under all three conditions, the amount of time required to reach this stage varied between conditions. To ensure that changes in protein quantity were due to growth condition and not growth time, we checked the quantity of proteins that can be affected by growth phase. For example, *recA* is responsible for DNA repair and is generally expressed in exponential phase; the amount decreases in stationary phase [13]. Also, proteins that are involved in transcription/translation are mostly expressed in the log phase of growth [14]. Of the proteins that we identified that are typically affected by growth phase, there were no significant changes in quantities of most of these (Table 2); therefore, we suggest that the quantitative changes we detected reflect growth condition effects and not changes due to growth pattern differences.

**Table 2:** *O. carboxidovorans* growth phase proteins identified in this study.

Locus tag	Protein name	Functional category
**Proteins with no statistically significant (**P>0.05**) changes**		
OCAR_6233	DNA gyrase A subunit	DNA metabolism
OCAR_5431	protein RecA	DNA metabolism
OCAR_6007	DNA topoisomerase IV B subunit	DNA metabolism
OCAR_5783	DNA topoisomerase 1	DNA metabolism
OCAR_4508	translation initiation factor IF-2	Protein synthesis
OCAR_5665	DNA-directed RNA polymerase	Protein synthesis
OCAR_6264	seryl-tRNA synthetase	Protein synthesis
OCAR_5680	ribosomal protein L2	Protein synthesis
OCAR_7287	tRNA(Ile)-lysidine synthase	Biosynthesis of cofactors, prosthetic groups, and carriers
**Proteins that had significantly (**P≤0.05**) changed expression in at least one comparison**		
OCAR_5689	50S ribosomal protein L5	Protein synthesis
OCAR_5955	ribosomal protein S2	Protein synthesis
OCAR_ 5701	DNA-directed RNA polymerase alpha subunit	Transcription
OCAR_5663	DNA-directed RNA polymerase beta subunit	Transcription
OCAR_4595	ATP synthase F1 beta subunit	Energy metabolism

### Carbon fixation and fatty acid biosynthesis

Because growth in syngas was associated with a shift in fatty acid profile by FAME analysis, we hypothesized that we would detect a correlation between expression of proteins in CO fixation and fatty acid biosynthesis proteins. Table 3 shows proteins expressed in the presence of syngas that are important for carbon fixation and fatty acid synthesis.

**Table 3:** Fatty acid metabolism and carbon fixation proteins expressed during growth in syngas.

Locus tag	Product name	GI	COG(s)
**Fatty acid metabolism**			
OCAR_4285	malonyl-CoA synthase	209871638	COG0318IQ
OCAR_6484	3-oxoacyl-[acyl-carrier-protein] synthase 2 (FabF)	209885605	COG0304IQ
**CO and H_2_ utilization**			
pHCG3_069	HoxL	47177023	NAΨ
pHCG3_033	CoxC	47176991	NA
pHCG3_041	CoxF	47176997	NA
pHCG3_72	Ni/Fe hydrogenase-like protein small subunit	47177026	NA

ΨNA. Not available.

The megaplasmid-encoded *cox* and *hox* operons proteins were expressed during growth in syngas. The *cox* genes enable CO utilization under aerobic chemolithoautotrophic conditions by catalyzing the oxidation of CO to CO_2_ [1], [15], [16]. CO dehydrogenase is the enzyme that catalyzes this process; it is an O_2_-stable, molybdenum-iron-sulfur-flavin hydroxylase that contains the molybdopterin cytosine dinucleotide-type molybdenum cofactor [17] and [2Fe-2S] centers of type I and type II [18], [19]. We detected CoxC, which is possibly part of a two component regulatory system and may play a role in CO sensing. CoxF was also detected; its function is not known, but it appears to contribute to posttranslational modification and maturation of the bimetallic [CuSMoO_2_]-center [1]. The *hox* genes encode a membrane-bound NiFeS-hydrogenase that oxidizes H_2_ and allows the bacteria to grow under an atmosphere of H_2_ [1], [15], [20], [21]. We detected HoxL, which is the large subunit of the hydrogenase.

The *O. carboxidovorans* OM5 genome has all the enzymes necessary for fatty acid biosynthesis (http://www.genome.jp/kegg-bin/show_pathway?org_name=oca&mapno=00061). FabF (3-oxoacyl-[acyl-carrier-protein] synthase 2), an important protein involved in the condensation process during fatty acid synthesis, was expressed in the presence of syngas. We also detected malonyl-CoA synthase in the presence of syngas. Fatty acid biosynthesis requires malonyl-CoA, which is a 3-C precursor that is transacylated to malonyl-acyl carrier protein (ACP) by malonyl-CoA acyl carrier protein transacylase [22]. Malonyl-CoA can be derived from acetyl-CoA by the action of acetyl CoA carboxylase; malonyl-CoA synthase allows bypass of the acetyl-CoA carboxylase reaction by synthesizing malonyl-CoA directly from malonate and coenyzyme A (Figure 3). In summary, although we detected some carbon fixation proteins and fatty acid biosynthesis proteins during growth in syngas, we could not definitively detect a correlation in their expression patterns due to growth conditions.

### TCA cycle and glyoxylate pathway

Acetate is a readily available form of fixed carbon that *O. carboxidovorans* can utilize for growth. During growth on acetate, the glyoxylate pathway would allow generation of acetyl CoA to enter fatty acid biosynthesis and gluconeogenesis. Isocitrate dehydrogenase, which is one of the TCA enzymes bypassed during the glyoxylate pathway, was decreased significantly in acetate medium compared to TSB, suggesting that the glyoxylate pathway is favored over the TCA cycle during growth on acetate. In *E. coli*, the flux between the TCA and glyoxylate cycles is controlled by modification of isocitrate dehydrogenase rather than on enzyme quantity [23]. However, in the more closely related species *Bradyrhizobium japonicum*, glyoxylate cycle flux is not controlled by covalent modification of isocitrate dehydrogenase enzyme [23]. We also detected an enzyme in the glyoxylate pathway and the TCA cycle (malate dehydrogenase) during growth in acetate and TSB.

### Proteins that increased expression in the syngas environment

Proteins that had significantly increased quantities when *O. carboxidovorans* was grown in the presence of syngas compared with growth in acetate medium and nutrient rich TSB medium are shown in Table S7. Some of these proteins are particularly remarkable in that they were increased in syngas compared with both heterotrophic growth conditions, so they are very likely to be important for growth under chemolithoautotrophic conditions.

#### (i) Cell envelope

Glycerophosphoryl diester phosphodiesterase was increased during growth in syngas. Glycerophosphodiesters are enzymatically produced by phospholipases A_1_ and A_2_ from membrane phospholipids [24]. These are further degraded by glycerophosphodiester phosphodiesterase, producing the corresponding alcohols and *sn*-glycerol-3-phosphate (G3P), which is an essential precursor for *de novo* synthesis of glycerophospholipids. Increased amounts of this enzyme involved in membrane phospholipid degradation may indicate altered fatty acid metabolism during growth in syngas, which could result in the changes in fatty acid composition in the cell envelope that we detected by FAME analysis. Further analysis by mutagenesis or dose-dependent studies is needed to confirm a relationship between this protein expression change and the changes in fatty acid composition.

Some cell wall metabolism enzymes increased during growth in syngas (Figure 3), including membrane-bound lytic murein transglycosylase A (MltA), which is involved in murein lysis; diaminopimelate epimerase, which is required for peptidoglycan and lysine biosynthesis; and peptidoglycan glycosyltransferase, which catalyzes the transglycosylation/transpeptidation crosslinking reaction in formation of the murein sacculus. Increased amounts of these cell wall enzymes may indicate increased turnover of peptidoglycan during growth in syngas.

#### (ii) Oxidative homeostasis

There was evidence that the syngas environment induced an increased need for *O. carboxidovorans* proteins involved in oxidative homeostasis. This is not surprising because *O. carboxidovorans* is deriving energy from the oxidation of CO and H_2_ during chemolithoautotrophic growth. For example, syngas induced increased riboflavin biosynthesis protein RibF, which is required for riboflavin synthesis. Riboflavin is a component of FMN and FAD, both of which are electron carriers important for oxidative homeostasis. Syngas also induced increased sulfite reductase relative to acetate medium. In addition to its sulfite reductase activity, this enzyme catalyzes the reversible reduction of flavins (riboflavin, FMN, and FAD) using NAD(P) as an electron donor [25]; therefore, it could serve to regenerate either oxidized or reduced forms of flavin coenzymes as needed during growth in syngas.

Syngas also induced proteins involved in oxidative stress response. SufB was increased; the SufBCD complex acts synergistically with SufE to stimulate the cysteine desulfurase activity of SufS and contributes to the assembly or repair of oxygen-labile iron-sulfur clusters under oxidative stress. Syngas was associated with increased NADH dehydrogenase (quinone) g subunit, which mediates resistance to the oxidative quinone menadione [26].

#### (iii) Regulatory proteins involved in signal transduction

Regulatory proteins may play a role in adapting *O. carboxidovorans* metabolism to chemolithoautotrophic conditions (Figure 3). One such protein is the nitrogen regulatory protein NtrY, which had increased in quantity in syngas. *O. carboxidovorans* is not a known nitrogen fixer, but it is closely related to *Nitrobacter hamburgensis* based on 16S rRNA-based phylogeny and on protein comparisons. Overall, *O. carboxidovorans* and *N. hamburgensis* share 1148 orthologous proteins (bidirectional best hits (BBHs) with identity ≥70% [27]). NtrY is unlikely to regulate nitrogen metabolism in *O. carboxidovorans*, but it may play a different regulatory role in this species. Diguanylate cyclase/phosphodiesterase also increased. This is a heme-β-containing sensor protein that may be involved in sensing and controlling changes from aerobic to anaerobic metabolism, and this protein exhibits phosphodiesterase (PDE) activity with c-di-GMP and cAMP. It might be required during growth in syngas because of the CO_2_ rich atmosphere and almost anaerobic conditions. Other regulatory proteins that increased in syngas included C4-dicarboxylate transport transcriptional regulatory protein and a multi-sensor hybrid histidine kinase.

#### (iv) Bacterial conjugation

TrbL was significantly increased in syngas, suggesting an active conjugation state. Conjugation proteins encoded on the *O. carboxidovorans* megaplasmid have ≥52% identity at the amino acid level with the corresponding proteins coding for the conjugation system on the Ti plasmid in *Agrobacterium tumefaciens.* The Ti plasmid is responsible for conjugative transmission of plant tumor formation by F-mediated conjugation. TraA is required for pilus biosynthesis during conjugal transfer [1]. TraI and TrbI proteins were also found to be expressed in the presence of syngas. The *O. carboxidovorans* megaplasmid encodes CO, CO_2_, and H_2_ utilization systems; therefore, the plasmid conjugation system is upregulated under environmental conditions where plasmid-encoded genes are being actively transcribed. Thus, exchange of the megaplasmid is more likely under environmental conditions where plasmid genes are being utilized.

#### (v) Transport proteins

Several transport proteins had increased quantities in syngas. Two ABC transporter system proteins were increased, including one involved in taurine uptake. Two efflux proteins were also increased, including CzcA, which has a low cation transport activity for cobalt. It is essential for cobalt, zinc, and cadmium resistance. CzcA may facilitate iron uptake from extracellular iron chelators under iron limitation, which may explain its upregulation in syngas. A multidrug resistance efflux protein was also upregulated.

#### (vi) Metabolic enzymes

Succinyl CoA synthetase was increased during growth in syngas relative to acetate medium, which is logical because this enzyme is part of the TCA cycle that is bypassed when the glyoxylate pathway is active during growth on acetate. Some biosynthetic proteins increased in quantity in minimal medium with syngas, which is expected because *O. carboxidovorans* would need to synthesize amino acids and cofactors under this growth condition. Examples include ATP phosphribosyltransferase, which is required for histidine biosynthesis; FolC, which is required for folate synthesis; and FeS assembly protein SufB. Some other metabolic enzymes that increased in quantity are involved in biosynthesis or degradation of metabolites that are not characterized well (for example, homospermidine synthase and 3-oxoadipate enol-lactonase), which simply reveals how little is understood of the metabolism of this environmental bacterium.

### Conclusions

An important characteristic of *O. carboxidovorans* is its ability to lead an autotrophic or a heterotrophic lifestyle to take advantage of its environment. This switch in metabolism is likely governed by several regulatory genes and sensor proteins. The current study identified some of these potential regulatory proteins and shows that chemolithoautotrophic growth induces important changes in *O. carboxidovorans* cell envelope composition and turnover, oxidation homeostasis, and other metabolic enzymes.

The other important and extraordinary characteristic of *O. carboxidovorans* is its ability to utilize CO and fix carbon from CO_2_. CO is highly toxic to respiring organisms even in low concentrations, and CO_2_ is the most abundant greenhouse gas. Thus, *O. carboxidovorans* has the ability to produce biodiesel components from unwanted gaseous carbon, and this may be a unique form of bacterial carbon sequestration. Production of biodiesel from syngas, itself produced by gasification of waste biomass, is an environment-friendly application. Further research is needed to optimize conditions to produce greater amounts of long chain fatty acids and scale up the process.

## Materials and Methods

### Growth conditions

For routine cultivation and for experimental nutrient rich growth, *Oligotropha carboxidovorans* OM5 (ATCC no. 49405) was grown in tryptic soy broth (TSB) in flasks at 30°C with rotary shaking. For growth under syngas and acetate conditions, *O. carboxidovorans* was grown in a minimal medium (carboxydobacterium medium; ATCC no 1789) supplemented with syngas or acetate to serve as a carbon source. For heterotrophic growth, sodium acetate (0.3%) was added to the flask containing autoclaved minimal medium. For growth in syngas (chemolithoautotrophic growth), *O. carboxidovorans* was inoculated in minimal medium and incubated in an atmosphere containing 50% air and 50% syngas (3% methane, 18% CO_2_, and 41% CO, with H_2_ making up the balance) using a New Brunswick 410 BioFlo 14 L fermentor continuously fed with syngas and oxygen.

**Figure 4:**
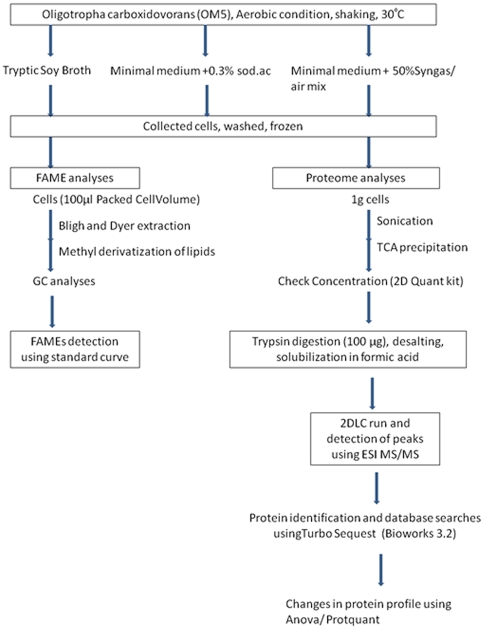
A flow diagram outlining the experimental workflow.

Three independent biological replicates were cultivated for each treatment at 30°C until they reached an optical density at 600 nm (OD_600_) of 0.6. Growth curves were plotted for each treatment. Bacteria were pelleted by centrifugation (5000×*g*), washed with phosphate buffer (0.1 M, pH 7), and quickly frozen at −80°C. Figure 4 outlines the experimental set up.

### Fatty acid methyl ester (FAME) analysis

Lipids were extracted from bacteria grown under each condition using modified Bligh and Dyer Extraction[28], followed by methyl derivatization of the lipids. A Varian Star 3400 gas chromatogram was used with helium as a carrier gas passing through a Resek Stable Wax DA column (30 m×0.25 mm, with a 0.25 mm film thickness) coupled with the Varian Saturn 2000 MS. This was equipped with wave board technology, and the NIST (National Institute of Standards and Technology) library was installed to allow for methyl ester identification. The amount of fatty acid was determined by comparing the means of two different analyses to a standard generated curve run [29], [30], [31].

### Proteomics

*O. carboxidovorans* pellets (1 *g*) were lysed in 100 mM Tris-Cl pH 8.0, 2% Triton X-100, 2.6 mg/ml sodium azide, 8 mM PMSF (phenylmethylsulfonyl fluoride) by sonication on ice (4 pulses of 15 s duration each). The supernatants were treated with 50% cold TCA (trichloroacetate), and the precipitated protein was washed with acetone. The pellets were resuspended in solubilization buffer (7 M urea, 20 mM tris-Cl, pH 8.0, 5 mM EDTA, 5 mM MgCl_2_, 4% CHAPS), and protein concentration was determined using the Plus One 2-D Quant Kit (Amersham) following the manufacturer's instructions. Four replicates for each treatment were prepared and stored at −80°C.

One hundred micrograms of each protein sample was resuspended in 0.1 M ammonium bicarbonate, 5% HPLC grade ACN, reduced in 5 mM DTT (dithiothreitol) (65°C, 5 min), alkylated in 10 mM iodoacetamide (30°C, 30 min), and then trypsin digested until there was no visible pellet (1∶50 w/w 37°C, 16 h). Peptides were desalted using a peptide microtrap (Michrom BioResources, Auburn, CA) and eluted using a 0.1% TFA, 95% ACN solution. Desalted peptides were dried in a vacuum centrifuge and resuspended in 20 µl of 0.1% formic acid.

Peptides were separated by strong cation exchange (SCX) liquid chromatography (LC) followed by reverse phase (RP) LC coupled directly in line with electrospray ionization (ESI) tandem mass spectrometry (MS/MS). 2DLC ESI MS/MS was done exactly as described [32]. All searches were done using TurboSEQUEST (Bioworks Browser 3.2; Thermo Electron). Mass spectra and tandem mass spectra were searched against all annotated proteins from strain OM5. Cysteine carbamidomethylation and methionine oxidation (single and double) were included in the search strategy. We used the reverse database functionality in Bioworks 3.2 and searched MS^2^ data against a reversed OM5 database using identical search criteria. Protein identifications have been submitted to PRoteomics IDEntifications (PRIDE) database [33], [34] (accession number 10011). PRIDE submission requirements are based on the proposed guidelines by proteomics standards initiative [35]. Unprocessed mass spectrometry data showing peptide identifications under the three growth conditions are also available in Tables S8, S9, and S10.

To determine significant changes in proteins between the control and treatment datasets, only proteins identified with at least three peptides in any dataset were included in further analysis. The Sequest ΣXcorr can be used as a variant of spectral counting as a for non-isotopic quantification in shotgun proteomics [32]. Because *O. carboxidovorans* growth was different under different conditions (see Results and Discussion), the ΣXcorr was normalized to account for differences incurred by different growth: the ΣXcorr (for the individual protein)/ΣXcorr (for all identified proteins in a dataset) was calculated. These normalized ΣXcorr values were used for calculating the significant differences in protein amounts by ANOVA followed by Benjamini-Hochberg correction [36] for multiple testing (P≤0.05) using *Protquant* [37]. To provide a comparative overview of the proteins that were differentially expressed, heat maps were created using Heatmap Builder [38]. Functional categories for the heat maps were assigned by JCVI Annotation Service, which uses Blast-extend-repraze (BER) searches and Hidden Markov Model (HMM) searches to predict function.

## Supporting Information

Figure S1 **Heat maps showing relative expression of differentially expressed proteins from Tables S1, S2, S3, S4, S6 (organized by functional category).** Red represents increased expression and green represents lower expression.
(PDF)

Table S1 **Proteins that significantly increased in acetate medium compared to minimal medium with syngas.**
(DOCX)

Table S2 **Proteins that significantly decreased in acetate medium compared to minimal medium with syngas.**
(DOCX)

Table S3 **Proteins that significantly increased in acetate medium compared to TSB.**
(DOCX)

Table S4 **Proteins that significantly decreased in acetate medium compared to TSB.**
(DOCX)

Table S5 **Proteins that significantly decreased in minimal medium with syngas compared to TSB**
(DOCX)

Table S6 **Proteins that significantly increased in minimal medium with syngas compared to TSB.**
(DOCX)

Table S7 **Proteins with significantly increased (P≤0.05) quantities in the presence of syngas compared to TSB and/or AC.**
(DOCX)

Table S8 **Peptide identifications from TSB (unprocessed mass spectrometry data).**
(XLS)

Table S9 **Peptide identifications from acetate medium (unprocessed mass spectrometry data).**
(XLS)

Table S10 **Peptide identifications from minimal medium with syngas (unprocessed mass spectrometry data).**
(XLS)
