# Supplementary material for: Proteome and Membrane Fatty Acid Analyses on Oligotropha carboxidovorans OM5 Grown under Chemolithoautotrophic and Heterotrophic Conditions
Source: PLoS One. 2011 Feb 28;6(2):e17111. doi: 10.1371/journal.pone.0017111 (PMC3046131; doi:10.1371/journal.pone.0017111)

Amino acid biosynthesis

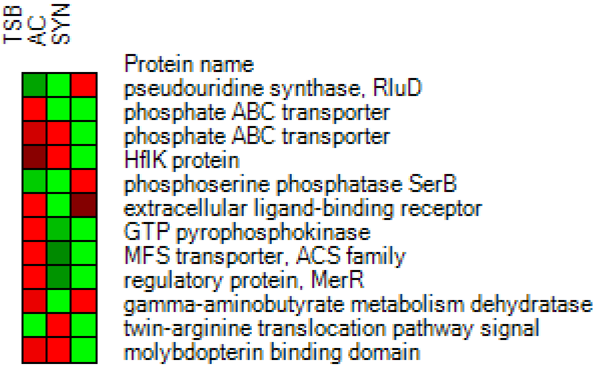

Biosynthesis of prosthetic groups, cofactors/carriers

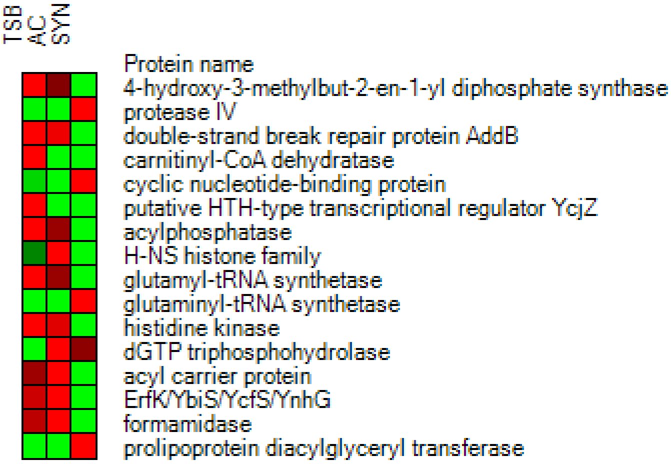

Cell envelope proteins

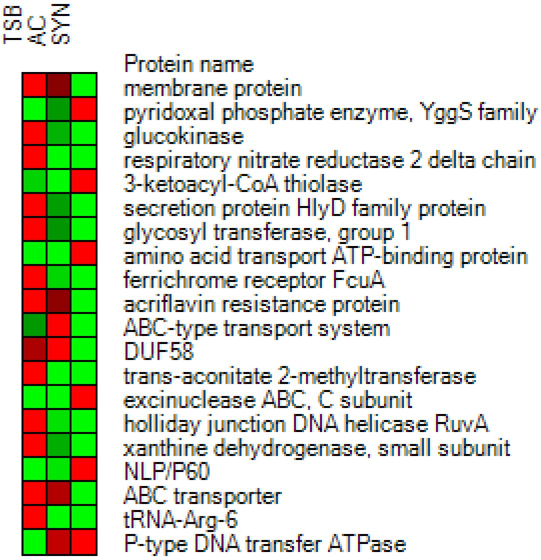

Central intermediary metabolism and cellular processes

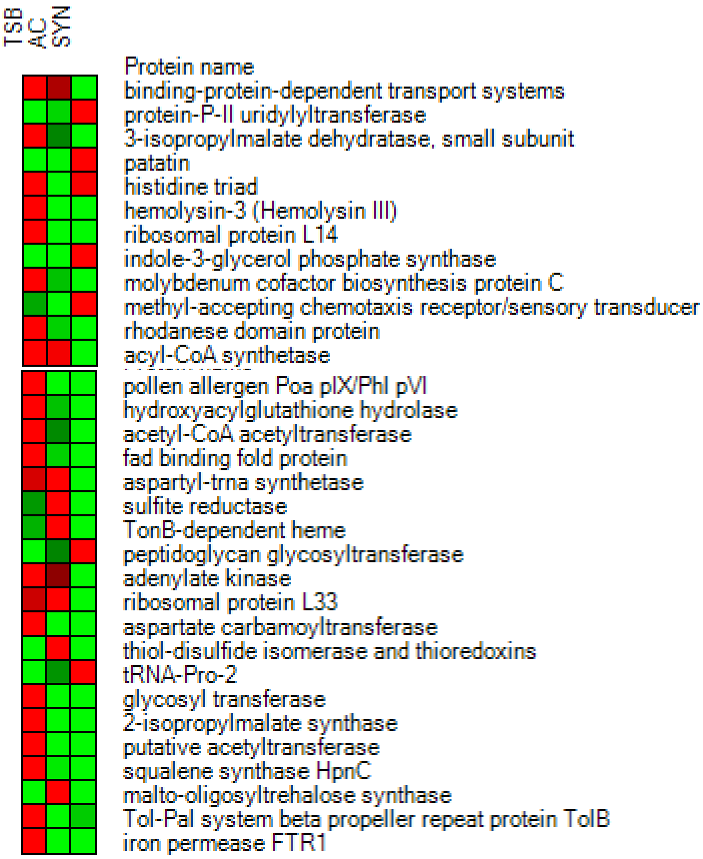

DNA metabolism

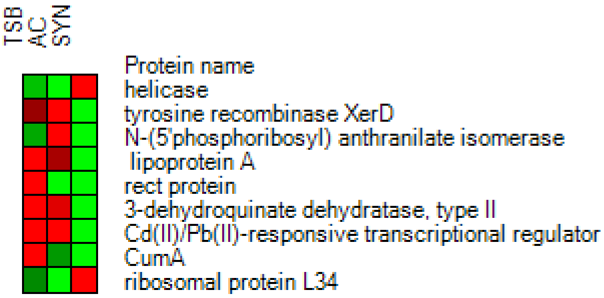

Energy metabolism

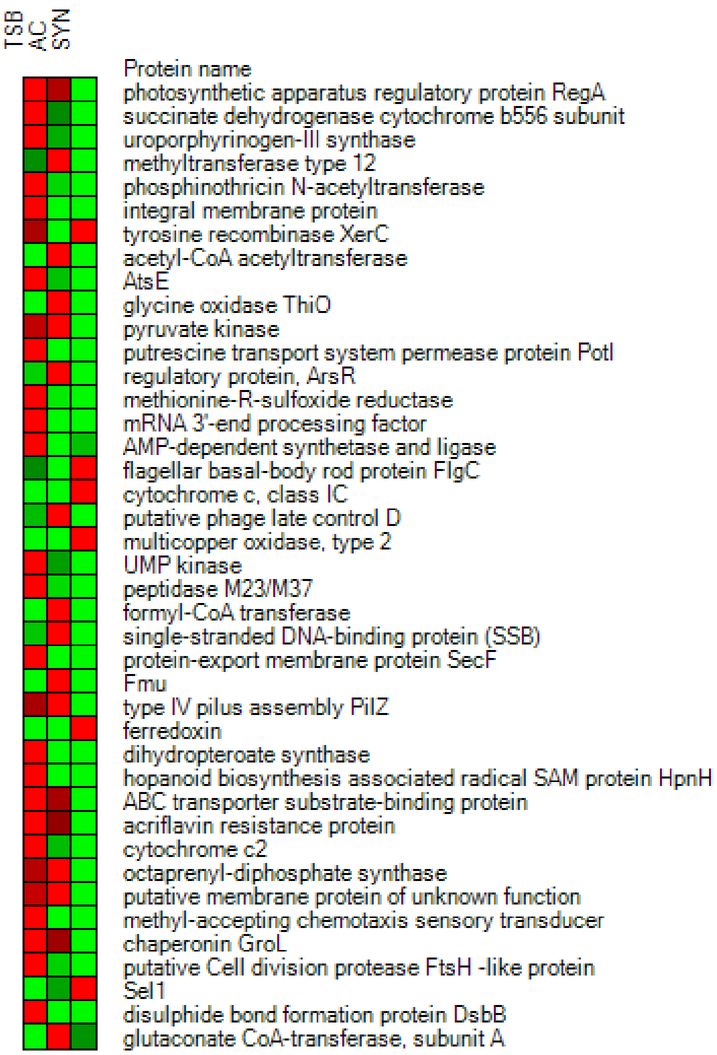

Fatty acid and phospholipid metabolism

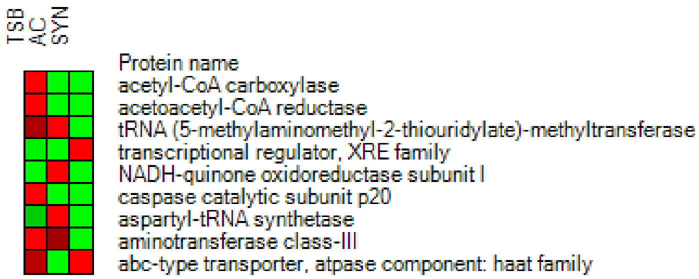

Protein synthesis

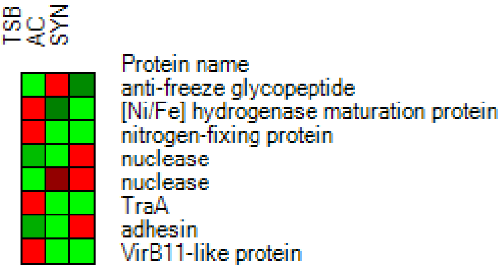

Purines, pyrimidines, nucleosides, nucletides, etc.

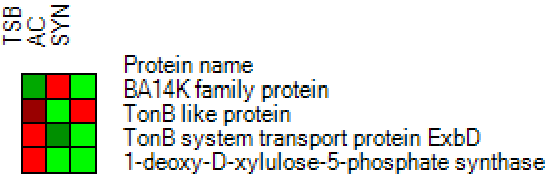

Protein fate

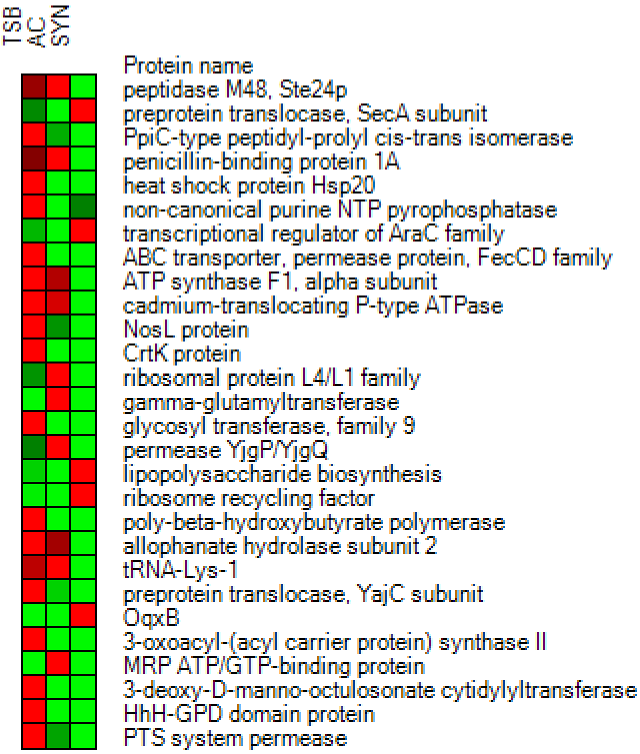

Regulatory functions

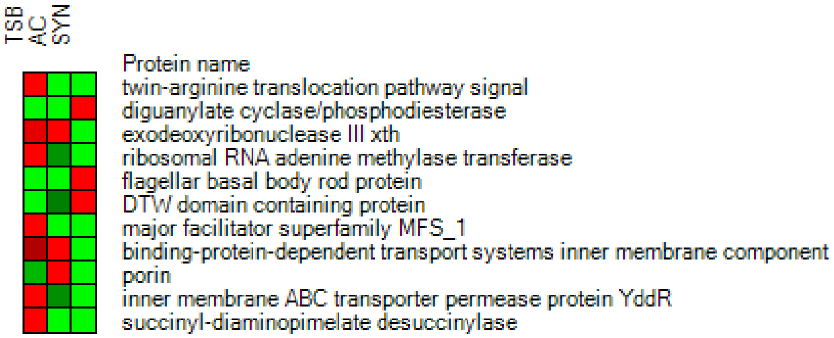

Transport and binding proteins

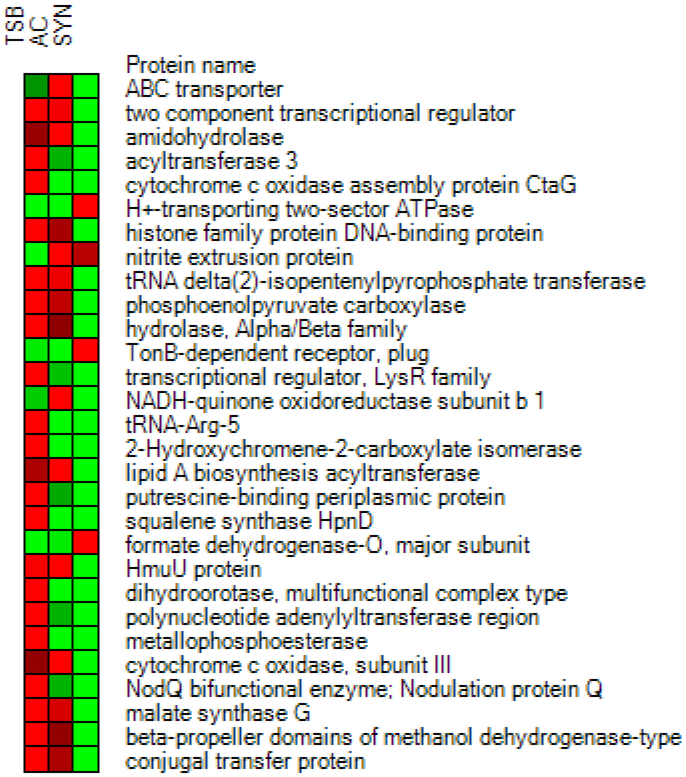

Transcription

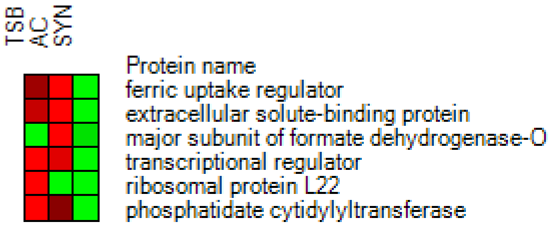

Unclassified protein or function unlisted

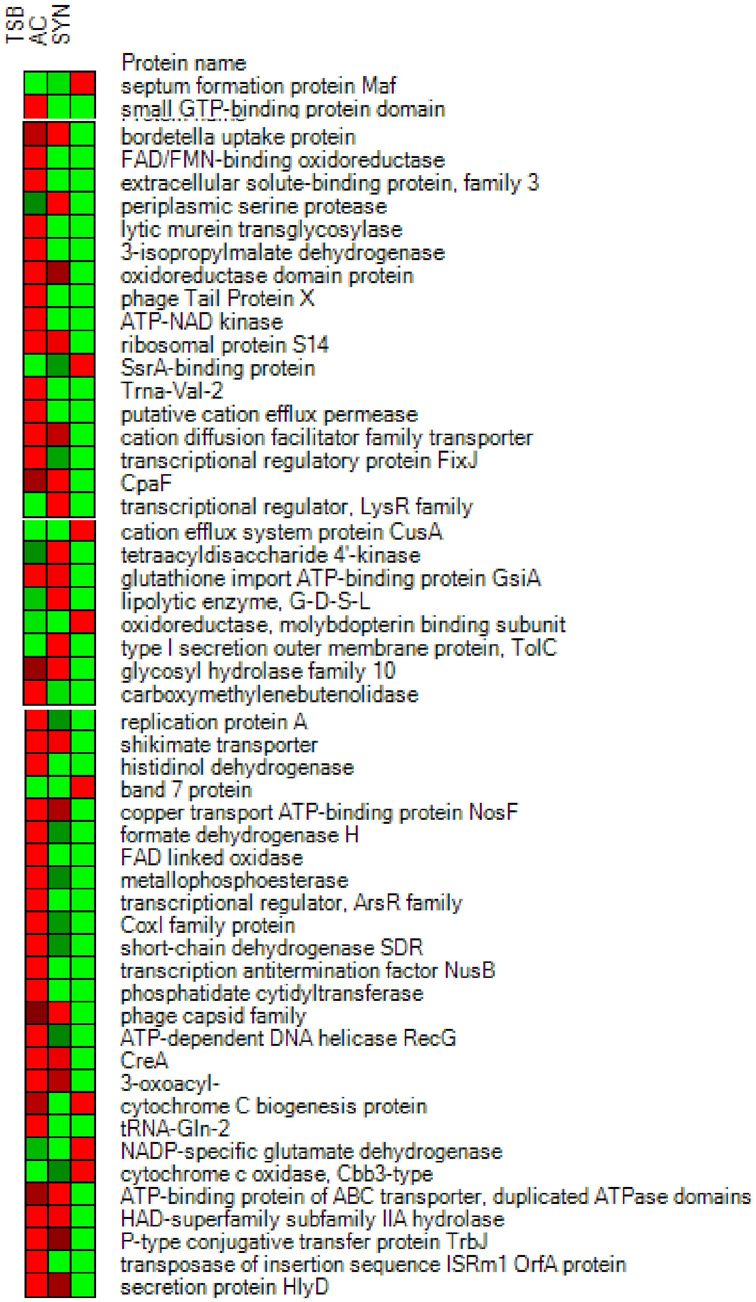

Hypothetical proteins

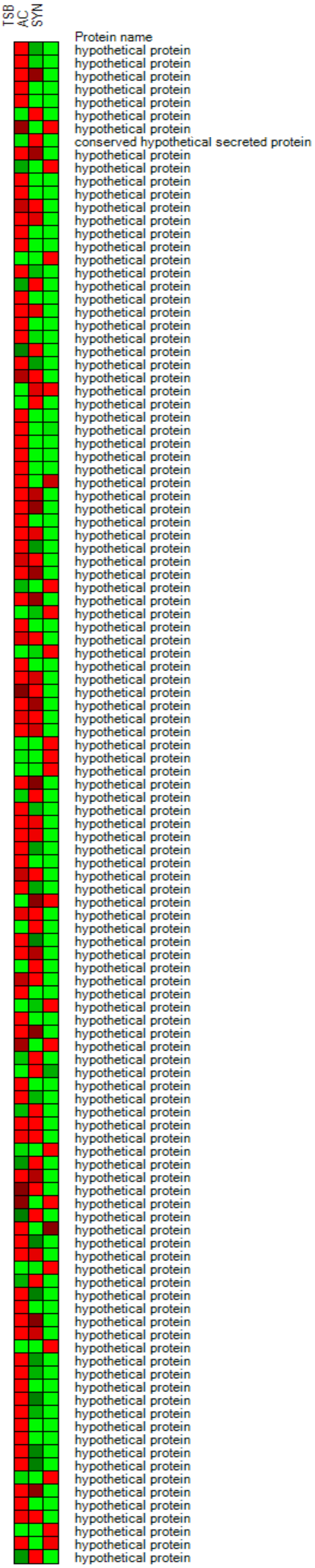

Supplement: Figure S1 — Heat maps showing relative expression of differentially expressed proteins from Tables S1, S2, S3, S4, S6 (organized by functional category). Red represents increased expression and green represents lower expression. (PDF) [file pone.0017111.s001.pdf]
