# Supplementary material for: Proteome and Membrane Fatty Acid Analyses on Oligotropha carboxidovorans OM5 Grown under Chemolithoautotrophic and Heterotrophic Conditions
Source: PLoS One. 2011 Feb 28;6(2):e17111. doi: 10.1371/journal.pone.0017111 (PMC3046131; doi:10.1371/journal.pone.0017111)
Supplement: Table S1 — Proteins that significantly increased in acetate medium compared to minimal medium with syngas. (DOCX) [file pone.0017111.s002.docx]

Table S1. Proteins that significantly increased in acetate medium compared to minimal medium with syngas

| *Locus tag* | *Protein name* | *% increase* | *Main role category* |
| --- | --- | --- | --- |
| OCAR_4263 | diaminopimelate epimerase | 100 | Amino acid biosynthesis |
| OCAR_5999 | FeS assembly protein SufB | 95.7 | Biosynthesis of cofactors, prosthetic groups, and carriers |
| OCAR_7232 | riboflavin biosynthesis protein RibF | 100 | Biosynthesis of cofactors, prosthetic groups, and carriers |
| OCAR_4480 | FolC bifunctional protein | 100 | Biosynthesis of cofactors, prosthetic groups, and carriers |
| OCAR_5052 | 2-dehydropantoate 2-reductase | 93.6 | Biosynthesis of cofactors, prosthetic groups, and carriers |
| OCAR_4064 | membrane protein Mlr2225 | 100 | Cell envelope |
| OCAR_6433 | membrane protein putative | 40.3 | Cell envelope |
| OCAR_5236 | peptidoglycan glycosyltransferase | 100 | Cell envelope |
| OCAR_5491 | dolichyl-phosphate beta-D-mannosyltransferase | 100 | Cell envelope |
| OCAR_7039 | membrane protein putative | 68.7 | Cell envelope |
| OCAR_7251 | tetraacyldisaccharide 4prime-kinase | 100 | Cell envelope |
| OCAR_4348 | tetraacyldisaccharide 4p-kinase | 100 | Cell envelope |
| OCAR_6064 | multidrug resistance protein MdtB (Multidrug transporter mdtB) | 57 | Cellular processes |
| OCAR_4912 | homospermidine synthase (HSS) | 100 | Central intermediary metabolism |
| OCAR_6338 | Ppx/GppA phosphatase | 100 | Central intermediary metabolism |
| OCAR_4879 | sulfite reductase | 93.8 | Central intermediary metabolism |
| OCAR_0131 | protein-P-II uridylyltransferase | 85.7 | Central intermediary metabolism |
| OCAR_3581 | DNA polymerase I (POL I) | 100 | DNA metabolism |
| OCAR_6675 | Fe-S oxidoreductase | 100 | Energy metabolism |
| OCAR_5483 | 3-oxoadipate enol-lactonase | 100 | Energy metabolism |
| OCAR_5286 | NADH dehydrogenase (quinone) g subunit | 100 | Energy metabolism |
| OCAR_6889 | methylmalonate-semialdehyde dehydrogenase (acylating) | 71 | Energy metabolism |
| OCAR_6554 | succinyl-CoA synthetase beta chain (SCS-alpha) | 83 | Energy metabolism |
| OCAR_4595 | ATP synthase F1 beta subunit | 46.7 | Energy metabolism |
| OCAR_5771 | glycerophosphoryl diester phosphodiesterase | 100 | Fatty acid and phospholipid metabolism |
| OCAR_7077 | thioesterase superfamily | 100 | Fatty acid and phospholipid metabolism |
| OCAR_7510 | Aryldialkylphosphatase | 100 | Fatty acid and phospholipid metabolism |
| OCAR_4023 | hypothetical protein | 100 | Hypothetical proteins |
| OCAR_4549 | hypothetical protein | 100 | Hypothetical proteins |
| OCAR_4778 | conserved hypothetical protein | 100 | Hypothetical proteins |
| OCAR_5614 | hypothetical protein | 100 | Hypothetical proteins |
| OCAR_4778 | conserved hypothetical protein | 100 | Hypothetical proteins |
| OCAR_5605 | conserved hypothetical protein | 94.1 | Hypothetical proteins |
| OCAR_7602 | conjugal transfer protein TrbL | 66 | Mobile and extrachromosomal element functions |
| OCAR_4057 | peptidase M48 Ste24p | 100 | Protein fate |
| OCAR_7426 | cytosol aminopeptidase (Leucine aminopeptidase) (LAP) | 100 | Protein fate |
| OCAR_6104 | methyltransferase type 11 | 78 | Protein synthesis |
| OCAR_7263 | ATP phosphoribosyltransferase (ATP-PRTase) (ATP-PRT) | 93 | Purines, pyrimidines, nucleosides, and nucleotides |
| OCAR_4110 | diguanylate cyclase/phosphodiesterase | 93 | Regulatory functions |
| OCAR_5401 | multi-sensor hybrid histidine kinase | 100 | Regulatory functions |
| OCAR_6097 | nitrogen regulation protein NtrY | 54 | Regulatory functions |
| OCAR_5665 | DNA-directed RNA polymerase betap subunit | 93 | Transcription |
| OCAR_5649 | taurine transport system permease protein TauC | 100 | Transport and binding proteins |
| OCAR_4705 | C4-dicarboxylate transport transcriptional regulatory protein DctR | 100 | Transport and binding proteins |
| OCAR_6437 | cation efflux system protein CzcA | 95.6 | Transport and binding proteins |
| OCAR_4899 | branched-chain amino acid ABC transporter permease protein | 93 | Transport and binding proteins |
| OCAR_4391 | MltA | 83 | Unclassified |
| OCAR_2305 | pANL56 | 100 | Unclassified |
| OCAR_4066 | bordetella uptake gene (bug) product superfamily | 90 | Unknown function |
