# Supplementary material for: Proteome and Membrane Fatty Acid Analyses on Oligotropha carboxidovorans OM5 Grown under Chemolithoautotrophic and Heterotrophic Conditions
Source: PLoS One. 2011 Feb 28;6(2):e17111. doi: 10.1371/journal.pone.0017111 (PMC3046131; doi:10.1371/journal.pone.0017111)
Supplement: Table S2 — Proteins that significantly decreased in acetate medium compared to minimal medium with syngas. (DOCX) [file pone.0017111.s003.docx]

Table S2. Proteins that significantly decreased in acetate medium compared to minimal medium with syngas

| *Locus tag* | *Protein name* | *% decrease* | *Main role category* |
| --- | --- | --- | --- |
| OCAR_5892 | O-acetylhomoserine (thiol)-lyase (O-acetylhomoserinesulfhydrylase) | 100 | Amino acid biosynthesis |
| OCAR_5292 | ketol-acid reductoisomerase | 94.4 | Amino acid biosynthesis |
| OCAR_6751 | 5-methyltetrahydropteroyltriglutamate--homocysteine S-methyltransferase | 100 | Amino acid biosynthesis |
| OCAR_6569 | glutamate-ammonia-ligase adenylyltransferase | 100 | Amino acid biosynthesis |
| OCAR_4938 | glutamate synthase [NADH] (nadh-gogat) | 100 | Amino acid biosynthesis |
| OCAR_4655 | ornithine carbamoyltransferase | 100 | Amino acid biosynthesis |
| OCAR_7049 | NADP-specific glutamate dehydrogenase (nadp-gdh) | 89 | Amino acid biosynthesis |
| OCAR_6532 | molybdenum cofactor biosynthesis protein A | 100 | Biosynthesis of cofactors, prosthetic groups, and carriers |
| OCAR_7126 | uroporphyrinogen decarboxylase | 100 | Biosynthesis of cofactors, prosthetic groups, and carriers |
| OCAR_7085 | 2-dehydropantoate 2-reductase | 100 | Biosynthesis of cofactors, prosthetic groups, and carriers |
| OCAR_5998 | cysteine desulfurase (Selenocysteine lyase) | 100 | Biosynthesis of cofactors, prosthetic groups, and carriers |
| OCAR_4349 | 2-octaprenyl-6-methoxyphenyl hydroxylase | 100 | Biosynthesis of cofactors, prosthetic groups, and carriers |
| OCAR_4490 | 2-polyprenylphenol 6-hydroxylase | 100 | Biosynthesis of cofactors, prosthetic groups, and carriers |
| OCAR_6388 | 3-methyl-2-oxobutanoate hydroxymethyltransferase | 100 | Biosynthesis of cofactors, prosthetic groups, and carriers |
| OCAR_5363 | membrane protein putative | 100 | Cell envelope |
| OCAR_6718 | OmpA domain protein transmembrane region-containing protein | 100 | Cell envelope |
| OCAR_6489 | PepSY-associated TM helix domain protein | 100 | Cell envelope |
| OCAR_6893 | histone deacetylase superfamily | 100 | Cell envelope |
| OCAR_5391 | lipoprotein putative | 100 | Cell envelope |
| OCAR_4293 | OmpA/MotB | 100 | Cell envelope |
| OCAR_5482 | membrane protein putative | 100 | Cell envelope |
| OCAR_4795 | lipoprotein putative | 100 | Cell envelope |
| OCAR_7164 | phosphoglucosamine mutase | 100 | Cell envelope |
| OCAR_4891 | antibiotic biosynthesis monooxygenase | 100 | Cellular processes |
| OCAR_4902 | superoxide dismutase [Mn] (General stress protein 24)(GSP24) | 100 | Cellular processes |
| OCAR_5736 | peroxiredoxin-6 | 100 | Cellular processes |
| OCAR_0907 | superoxide dismutase [Mn] | 88.6 | Cellular processes |
| OCAR_4107 | acetyl-CoA acetyltransferase (Acetoacetyl-CoA thiolase) | 100 | Cellular processes |
| OCAR_5970 | methyl-accepting chemotaxis sensory transducer with Pas/Pac sensor | 100 | Cellular processes |
| OCAR_7144 | copper-translocating P-type ATPase | 100 | Cellular processes |
| OCAR_7105 | flagellar biosynthesis protein FlhA | 100 | Cellular processes |
| OCAR_6898 | methyl-accepting chemotaxis sensory transducer | 100 | Cellular processes |
| OCAR_4500 | Catalase | 87.5 | Cellular processes |
| OCAR_5319 | methionine adenosyltransferase | 100 | Central intermediary metabolism |
| OCAR_6096 | nitrogen regulation protein NR(I) | 100 | Central intermediary metabolism |
| OCAR_4608 | Acetyltransferase | 100 | Central intermediary metabolism |
| OCAR_7097 | short-chain dehydrogenase/reductase SDR | 100 | Central intermediary metabolism |
| OCAR_7643 | replication protein C | 100 | DNA metabolism |
| OCAR_5565 | C-5 cytosine-specific DNA methylase | 100 | DNA metabolism |
| OCAR_4483 | double-strand break repair protein AddB | 100 | DNA metabolism |
| OCAR_4841 | gene transfer agent | 100 | DNA metabolism |
| OCAR_4078 | tyrosine recombinase XerD | 100 | DNA metabolism |
| OCAR_5774 | DNA polymerase IV (Pol IV) | 100 | DNA metabolism |
| OCAR_5070 | Transaldolase | 100 | Energy metabolism |
| OCAR_5440 | isocitrate dehydrogenase NADP-dependent | 100 | Energy metabolism |
| OCAR_6560 | 2-hydroxy-3-oxopropionate reductase | 100 | Energy metabolism |
| OCAR_7074 | methylisocitrate lyase | 100 | Energy metabolism |
| OCAR_0332 | acetate--CoA ligase | 100 | Energy metabolism |
| OCAR_5095 | NAD(P) transhydrogenase subunit alpha | 100 | Energy metabolism |
| OCAR_7076 | 2-methylcitrate dehydratase 2 | 95 | Energy metabolism |
| OCAR_5971 | citrate (Si)-synthase | 89.3 | Energy metabolism |
| OCAR_5003 | citrate utilization protein B | 100 | Energy metabolism |
| OCAR_6099 | D-alanine aminotransferase (d-aspartateaminotransferase) | 100 | Energy metabolism |
| OCAR_5613 | Nitroreductase | 100 | Energy metabolism |
| OCAR_4571 | aconitate hydratase 1 | 100 | Energy metabolism |
| OCAR_6502 | alcohol dehydrogenase | 100 | Energy metabolism |
| OCAR_6994 | Carboxymethylenebutenolidase | 100 | Energy metabolism |
| OCAR_7310 | Transketolase | 100 | Energy metabolism |
| OCAR_5472 | cytochrome c biogenesis protein transmembrane region | 100 | Energy metabolism |
| OCAR_6723 | pkhd-type hydroxylase Blr3905 | 100 | Energy metabolism |
| OCAR_6529 | ribose 5-phosphate isomerase A | 100 | Energy metabolism |
| OCAR_5045 | respiratory nitrate reductase 2 delta chain | 100 | Energy metabolism |
| OCAR_4582 | oxoglutarate dehydrogenase (succinyl-transferring) E1 component | 100 | Energy metabolism |
| OCAR_6558 | pyruvate kinase | 89 | Energy metabolism |
| OCAR_5149 | NADPH-dependent fmn reductase | 100 | Energy metabolism |
| OCAR_7313 | fructose-bisphosphate aldolase class-I | 100 | Energy metabolism |
| OCAR_5030 | nitrous-oxide reductase | 100 | Energy metabolism |
| OCAR_7336 | propionate--CoA ligase (Propionyl-CoA synthetase) | 100 | Energy metabolism |
| OCAR_5329 | UDP-glucose 4-epimerase | 100 | Energy metabolism |
| OCAR_4995 | carnitinyl-CoA dehydratase (Crotonobetainyl-CoAhydratase) | 100 | Fatty acid and phospholipid metabolism |
| OCAR_6366 | acyl carrier protein | 100 | Fatty acid and phospholipid metabolism |
| OCAR_5960 | phosphatidate cytidylyltransferase | 100 | Fatty acid and phospholipid metabolism |
| OCAR_2510 | beta-ketoacyl synthase | 100 | Fatty acid and phospholipid metabolism |
| OCAR_7737 | conserved hypothetical protein | 100 | Hypothetical proteins |
| OCAR_6144 | conserved hypothetical protein | 100 | Hypothetical proteins |
| OCAR_5359 | conserved hypothetical protein | 99 | Hypothetical proteins |
| OCAR_5293 | conserved hypothetical protein | 100 | Hypothetical proteins |
| OCAR_6364 | conserved hypothetical protein | 100 | Hypothetical proteins |
| OCAR_4715 | conserved hypothetical protein | 100 | Hypothetical proteins |
| OCAR_7182 | conserved hypothetical protein | 100 | Hypothetical proteins |
| OCAR_5737 | conserved hypothetical protein | 100 | Hypothetical proteins |
| OCAR_7400 | conserved hypothetical protein | 100 | Hypothetical proteins |
| OCAR_5605 | conserved hypothetical protein | 100 | Hypothetical proteins |
| OCAR_5021 | conserved hypothetical protein | 100 | Hypothetical proteins |
| OCAR_7649 | phage integrase | 100 | Mobile and extrachromosomal element functions |
| OCAR_5530 | phage integrase | 100 | Mobile and extrachromosomal element functions |
| OCAR_5767 | ATP-dependent Clp protease ATP-binding subunit ClpA | 100 | Protein fate |
| OCAR_4083 | peptidyl-prolyl cis-trans isomerase cyclophilin-type | 100 | Protein fate |
| OCAR_4896 | twin-arginine translocation pathway signal | 98 | Protein fate |
| OCAR_4130 | penicillin-binding protein 1A (PBP-1a) (PBP1a) | 100 | Protein fate |
| OCAR_6288 | twin-arginine translocation pathway signal | 100 | Protein fate |
| OCAR_6256 | ATP-dependent protease subunit | 100 | Protein fate |
| OCAR_5697 | preprotein translocase SecY subunit | 100 | Protein fate |
| OCAR_4057 | peptidase M48 Ste24p | 100 | Protein fate |
| OCAR_7422 | CpaF | 100 | Protein fate |
| OCAR_6572 | protease Do subfamily | 100 | Protein fate |
| OCAR_5908 | Clp protease | 100 | Protein fate |
| OCAR_6260 | peptidase M23B | 100 | Protein fate |
| OCAR_6955 | peptidase S49 | 100 | Protein fate |
| OCAR_4267 | signal recognition particle protein | 90 | Protein fate |
| OCAR_5502 | chaperonin GroL | 100 | Protein fate |
| OCAR_5674 | translation elongation factor G | 100 | Protein synthesis |
| OCAR_6390 | peptide chain release factor 3 | 100 | Protein synthesis |
| OCAR_5207 | tRNA (5-methylaminomethyl-2-thiouridylate)-methyltransferase | 100 | Protein synthesis |
| OCAR_6374 | ribosomal protein L9 | 100 | Protein synthesis |
| OCAR_6949 | glycyl-tRNA synthetase beta subunit | 100 | Protein synthesis |
| OCAR_5435 | alanyl-tRNA synthetase | 100 | Protein synthesis |
| OCAR_7469 | glutamyl-tRNA(Gln) amidotransferase subunit A (Glu-ADTsubunit A) | 100 | Protein synthesis |
| OCAR_4370 | tRNA uridine 5-carboxymethylaminomethyl modification enzyme GidA | 100 | Protein synthesis |
| OCAR_6472 | ribosomal RNA large subunit methyltransferase J | 100 | Protein synthesis |
| OCAR_6125 | methionyl-tRNA synthetase | 87.5 | Protein synthesis |
| OCAR_6346 | nucleoside diphosphate kinase (NDK) (NDP kinase) | 100 | Purines, pyrimidines, nucleosides, and nucleotides |
| OCAR_7201 | two-component hybrid sensor and regulator | 100 | Regulatory functions |
| OCAR_4739 | membrane protein involved in aromatic hydrocarbon degradation | 100 | Regulatory functions |
| OCAR_6443 | type IV secretory pathway VirD2 components | 100 | Regulatory functions |
| OCAR_7133 | transcriptional regulatory protein FixJ | 100 | Regulatory functions |
| OCAR_5079 | transcriptional regulatory protein LysR family | 100 | Regulatory functions |
| OCAR_4994 | transcriptional regulator IclR family | 100 | Regulatory functions |
| OCAR_5985 | ribonuclease E (RNase E) | 100 | Transcription |
| OCAR_4503 | polyribonucleotide nucleotidyltransferase | 100 | Transcription |
| OCAR_5130 | transcription elongation factor GreA | 100 | Transcription |
| OCAR_5657 | transcription termination/antitermination factor NusG | 100 | Transcription |
| OCAR_5663 | DNA-directed RNA polymerase beta subunit | 100 | Transcription |
| OCAR_5949 | lipoprotein-releasing system ATP-binding protein LolD | 100 | Transport and binding proteins |
| OCAR_6880 | ABC-type nitrate/sulfonate/bicarbonate transport systems periplasmic | 100 | Transport and binding proteins |
| OCAR_6480 | extracellular ligand-binding receptor | 100 | Transport and binding proteins |
| OCAR_6206 | extracellular solute-binding protein family 5 | 100 | Transport and binding proteins |
| OCAR_7386 | thiosulfate-binding protein | 100 | Transport and binding proteins |
| OCAR_6184 | extracellular solute-binding protein family 1 | 100 | Transport and binding proteins |
| OCAR_6537 | periplasmic mannitol-binding protein | 100 | Transport and binding proteins |
| OCAR_4901 | ABC transporter substrate-binding protein aliphatic sulphonates | 100 | Transport and binding proteins |
| OCAR_5297 | C4-dicarboxylate-binding periplasmic protein | 91 | Transport and binding proteins |
| OCAR_7431 | bacterial extracellular solute-binding proteins family 5 | 100 | Transport and binding proteins |
| OCAR_6478 | MFS permease | 100 | Transport and binding proteins |
| OCAR_7637 | lead cadmium zinc and mercury-transporting ATPase | 100 | Transport and binding proteins |
| OCAR_7290 | Tol-Pal system beta propeller repeat protein TolB | 100 | Transport and binding proteins |
| OCAR_4721 | ferrichrome receptor FcuA | 100 | Transport and binding proteins |
| OCAR_5632 | TonB-dependent receptor plug | 100 | Transport and binding proteins |
| OCAR_5867 | V-type H(+)-translocating pyrophosphatase | 100 | Transport and binding proteins |
| OCAR_6187 | cation diffusion facilitator family transporter | 100 | Transport and binding proteins |
| OCAR_6436 | cadmium-translocating P-type ATPase | 100 | Transport and binding proteins |
| OCAR_5667 | ABC transporter | 100 | Transport and binding proteins |
| OCAR_5650 | taurine import ATP-binding protein TauB 2 | 100 | Transport and binding proteins |
| OCAR_6722 | ExbB | 100 | Transport and binding proteins |
| OCAR_6041 | ABC transporter HlyB/MsbA family | 100 | Transport and binding proteins |
| OCAR_5477 | high-affinity branched-chain amino acid transport ATP-binding protein | 100 | Transport and binding proteins |
| OCAR_4066 | bordetella uptake gene (bug) product superfamily | 100 | Unknown function |
| OCAR_6219 | glycoside hydrolase family 17 | 100 | Unknown function |
| OCAR_5088 | peptidoglycan-binding domain 1 | 100 | Unknown function |
| OCAR_6409 | AAA ATPase central domain protein | 100 | Unknown function |
| OCAR_5436 | UspA | 100 | Unknown function |
| OCAR_7541 | RmuC domain protein | 100 | Unknown function |
| OCAR_4841 | putative homologue of Rhodobacter capsulatus gene transfer agent | 100 | Unknown function |
| OCAR_4736 | ggdef domain | 100 | Unknown function |
| OCAR_7461 | LemA | 100 | Unknown function |
| OCAR_6672 | squalene-associated FAD-dependent desaturase | 100 | Unknown function |
| OCAR_4038 | hypothetical protein | 100 | Hypothetical protein |
| OCAR_4345 | hypothetical protein | 100 | Hypothetical protein |
| OCAR_5915 | hypothetical protein | 100 | Hypothetical protein |
| OCAR_6039 | hypothetical protein | 100 | Hypothetical protein |
| OCAR_5528 | hypothetical protein | 100 | Hypothetical protein |
| OCAR_4734 | hypothetical protein | 100 | Hypothetical protein |
