# Supplementary material for: Proteome and Membrane Fatty Acid Analyses on Oligotropha carboxidovorans OM5 Grown under Chemolithoautotrophic and Heterotrophic Conditions
Source: PLoS One. 2011 Feb 28;6(2):e17111. doi: 10.1371/journal.pone.0017111 (PMC3046131; doi:10.1371/journal.pone.0017111)
Supplement: Table S3 — Proteins that significantly increased in acetate medium compared to TSB. (DOCX) [file pone.0017111.s004.docx]

Table S3. Proteins that significantly increased in acetate medium compared to TSB

|  | *Protein name* | *% increase* | *Main role category* |
| --- | --- | --- | --- |
| OCAR_6569 | glutamate-ammonia-ligase adenylyltransferase | 100 | Amino acid biosynthesis |
| OCAR_7049 | NADP-specific glutamate dehydrogenase (nadp-gdh) | 44.4 | Amino acid biosynthesis |
| OCAR_6307 | molybdopterin biosynthesis protein | 83 | Biosynthesis of cofactors, prosthetic groups, and carriers |
| OCAR_4679 | glutamate--cysteine ligase | 66.7 | Biosynthesis of cofactors, prosthetic groups, and carriers |
| Ocar_4123 | membrane protein putative | 87 | Cell envelope |
| OCAR_7736 | putative exported protein of unknown function | 22 | Cell envelope |
| OCAR_5736 | peroxiredoxin-6 | 30 | Cellular processes |
| OCAR_4502 | superoxide dismutase [Mn] | 20 | Cellular processes |
| OCAR_5250 | cell division protein FtsZ | 100 | Cellular processes |
| OCAR_4891 | antibiotic biosynthesis monooxygenase | 33 | Cellular processes |
| OCAR_5970 | methyl-accepting chemotaxis sensory transducer with Pas/ | 100 | Cellular processes |
| OCAR_7105 | flagellar biosynthesis protein FlhA | 100 | Cellular processes |
| OCAR_6064 | multidrug resistance protein MdtB (Multidrug transporter | 40 | Cellular processes |
| OCAR_4455 | 3p-5p exonuclease | 80 | DNA metabolism |
| OCAR_4841 | gene transfer agent | 100 | DNA metabolism |
| OCAR_7393 | malate synthase G | 75 | Energy metabolism |
| OCAR_6099 | D-alanine aminotransferase (d-aspartateaminotransferase) | 100 | Energy metabolism |
| OCAR_5003 | citrate utilization protein B | 100 | Energy metabolism |
| OCAR_5613 | Nitroreductase | 50 | Energy metabolism |
| OCAR_6502 | alcohol dehydrogenase | 100 | Energy metabolism |
| OCAR_5045 | respiratory nitrate reductase 2 delta chain | 100 | Energy metabolism |
| OCAR_6565 | succinate-CoA ligase alpha subunit subfamily | 100 | Energy metabolism |
| OCAR_6573 | cytochrome c biogenesis protein transmembrane region | 75 | Energy metabolism |
| OCAR_5149 | NADPH-dependent fmn reductase | 75 | Energy metabolism |
| OCAR_6366 | acyl carrier protein | 60 | Fatty acid and phospholipid metabolism |
| OCAR_5960 | phosphatidate cytidylyltransferase | 100 | Fatty acid and phospholipid metabolism |
| OCAR_5336 | conserved hypothetical protein | 64.6 | Hypothetical proteins |
| OCAR_7137 | conserved hypothetical protein | 100 | Hypothetical proteins |
| OCAR_7602 | conjugal transfer protein TrbL | 50 | Mobile and extrachromosomal element functions |
| OCAR_6166 | phage prohead protease HK97 family | 100 | Mobile and extrachromosomal element functions |
| OCAR_7649 | phage integrase | 100 | Mobile and extrachromosomal element functions |
| OCAR_5767 | ATP-dependent Clp protease ATP-binding subunit ClpA | 100 | Protein fate |
| OCAR_6572 | protease Do subfamily | 100 | Protein fate |
| OCAR_6955 | peptidase S49 | 100 | Protein fate |
| OCAR_5697 | preprotein translocase SecY subunit | 33 | Protein fate |
| OCAR_5675 | translation elongation factor Tu | 7.5 | Protein synthesis |
| OCAR_6390 | peptide chain release factor 3 | 100 | Protein synthesis |
| OCAR_5207 | tRNA (5-methylaminomethyl-2-thiouridylate)-methyltransferase | 14 | Protein synthesis |
| OCAR_6472 | ribosomal RNA large subunit methyltransferase J | 100 | Protein synthesis |
| OCAR_5689 | 50S ribosomal protein L5 | 100 | Protein synthesis |
| OCAR_6097 | nitrogen regulation protein NtrY | 40 | Regulatory functions |
| OCAR_4931 | diguanylate cyclase | 50 | Regulatory functions |
| OCAR_4739 | membrane protein involved in aromatic hydrocarbon degradation | 40 | Regulatory functions |
| OCAR_5170 | dead/deah box helicase | 28.6 | Transcription |
| OCAR_5949 | lipoprotein-releasing system ATP-binding protein LolD | 75 | Transport and binding proteins |
| OCAR_7386 | thiosulfate-binding protein | 5.6 | Transport and binding proteins |
| OCAR_5053 | hemin import ATP-binding protein HmuV | 10 | Transport and binding proteins |
| OCAR_6478 | MFS permease | 66.7 | Transport and binding proteins |
| OCAR_4901 | ABC transporter substrate-binding protein aliphatic subunit | 20.7 | Transport and binding proteins |
| OCAR_5854 | RDD | 100 | Unknown function |
| OCAR_7541 | RmuC domain protein | 100 | Unknown function |
| gi\|47176979 | anti-freeze glycopeptide | 100 | Unknown function |
| gi\|47177060 | nuclease [Oligotropha carboxidovorans] | 100 | Unknown function |
| OCAR_6043 | hypothetical protein | 100 | Unknown function |
| OCAR_5915 | hypothetical protein | 25 | Unknown function |
| OCAR_5528 | hypothetical protein | 60 | Unknown function |
