# Supplementary material for: Proteome and Membrane Fatty Acid Analyses on Oligotropha carboxidovorans OM5 Grown under Chemolithoautotrophic and Heterotrophic Conditions
Source: PLoS One. 2011 Feb 28;6(2):e17111. doi: 10.1371/journal.pone.0017111 (PMC3046131; doi:10.1371/journal.pone.0017111)
Supplement: Table S4 — Proteins that significantly decreased in acetate medium compared to TSB. (DOCX) [file pone.0017111.s005.docx]

Table S4. Proteins that significantly decreased in acetate medium compared to TSB

| *Locus tag* | *Protein name* | *% decrease* | *Main role category* |
| --- | --- | --- | --- |
| OCAR_7439 | methionine synthase | 100 | Amino acid biosynthesis |
| OCAR_4760 | argininosuccinate lyase | 88. 9 | Amino acid biosynthesis |
| OCAR_5860 | serine hydroxymethyltransferase | 84.7 | Amino acid biosynthesis |
| OCAR_4654 | acetylornithine aminotransferase | 100 | Amino acid biosynthesis |
| OCAR_5830 | dihydrodipicolinate synthase | 100 | Amino acid biosynthesis |
| OCAR_6225 | cysteine synthase A | 100 | Amino acid biosynthesis |
| OCAR_5892 | O-acetylhomoserine (thiol)-lyase | 62.5 | Amino acid biosynthesis |
| OCAR_6751 | methyltetrahydropteroyltriglutama | 68.6 | Amino acid biosynthesis |
| OCAR_5296 | 2-isopropylmalate synthase | 100 | Amino acid biosynthesis |
| OCAR_6278 | dihydroxy-acid dehydratase | 100 | Amino acid biosynthesis |
| OCAR_4263 | diaminopimelate epimerase | 100 | Amino acid biosynthesis |
| OCAR_6061 | cysteine synthase | 100 | Amino acid biosynthesis |
| OCAR_4283 | 3-isopropylmalate dehydrogenase | 66. 7 | Amino acid biosynthesis |
| OCAR_5746 | gamma-glutamyltransferase | 100 | Biosynthesis of cofactors, prosthetic groups, and carriers |
| OCAR_7000 | coproporphyrinogen III oxidase | 100 | Biosynthesis of cofactors, prosthetic groups, and carriers |
| OCAR_5007 | FO synthase | 100 | Biosynthesis of cofactors, prosthetic groups, and carriers |
| OCAR_7258 | 4-hydroxybenzoate polyprenyl transferase | 100 | Biosynthesis of cofactors, prosthetic groups, and carriers |
| OCAR_6237 | outer-membrane immunogenic protein | 88. 9 | Cell envelope |
| OCAR_7339 | immunogenic protein | 91 | Cell envelope |
| OCAR_7134 | response regulator receiver | 100 | Cell envelope |
| OCAR_6547 | membrane protein putative | 100 | Cell envelope |
| OCAR_5056 | negative regulator of AmpC AmpD | 100 | Cell envelope |
| OCAR_7082 | cellulose synthesis regulatory protein | 100 | Cell envelope |
| OCAR_5977 | response regulator receiver | 75 | Cell envelope |
| OCAR_7565 | udp-glucose 6-dehydrogenase | 100 | Cell envelope |
| OCAR_4551 | glucose-1-phosphate thymidylyltransferase | 100 | Cell envelope |
| OCAR_4619 | glucose-1-phosphate cytidylyltransferase | 75 | Cell envelope |
| OCAR_6551 | oxalyl-CoA decarboxylase | 100 | Cellular processes |
| OCAR_6708 | extracellular tungstate binding protein | 77.8 | Cellular processes |
| OCAR_7328 | hybrid peroxiredoxin hyPrx5 | 100 | Cellular processes |
| OCAR_4500 | Catalase | 85.8 | Cellular processes |
| OCAR_6550 | formyl-CoA transferase | 100 | Cellular processes |
| OCAR_5733 | S-(hydroxymethyl)glutathione dehydrogenase | 84.7 | Cellular processes |
| OCAR_5809 | methyl-accepting chemotaxis sensory | 100 | Cellular processes |
| OCAR_7754 | mercuric resistance operon regulator | 100 | Cellular processes |
| OCAR_4080 | pollen allergen Poa pIX/Phl pVI C- | 100 | Cellular processes |
| OCAR_4107 | acetyl-CoA acetyltransferase | 61. 6 | Cellular processes |
| OCAR_6080 | methyl-accepting chemotaxis sensory | 100 | Cellular processes |
| OCAR_4102 | hydroxyacylglutathione hydrolase | 80 | Cellular processes |
| OCAR_4912 | homospermidine synthase (HSS) | 100 | Central intermediary metabolism |
| OCAR_7088 | formamidase | 88.3 | Central intermediary metabolism |
| OCAR_6744 | sulfoacetaldehyde acetyltransferase | 85.8 | Central intermediary metabolism |
| OCAR_5706 | zinc-binding dehydrogenase | 100 | Central intermediary metabolism |
| OCAR_6562 | glyoxylate carboligase | 100 | Central intermediary metabolism |
| OCAR_4548 | sulfite reductase (NADPH) hemoprotein | 100 | Central intermediary metabolism |
| OCAR_6339 | polyphosphate kinase | 80 | Central intermediary metabolism |
| OCAR_5074 | haloacid dehalogenase type II | 100 | Central intermediary metabolism |
| OCAR_6763 | FAD-dependent pyridine nucleotide-dehydrogenase | 100 | Central intermediary metabolism |
| OCAR_7567 | DNA polymerase I (POL I) | 100 | DNA metabolism |
| OCAR_4841 | gene transfer agent | 100 | DNA metabolism |
| OCAR_5556 | DNA methylase N-4/N-6 domain protein | 100 | DNA metabolism |
| OCAR_4061 | Helicase | 100 | DNA metabolism |
| OCAR_5043 | nitrate reductase alpha subunit | 100 | Energy metabolism |
| OCAR_5177 | sulfur oxidation protein | 100 | Energy metabolism |
| OCAR_4684 | cytochrome c oxidase subunit II | 100 | Energy metabolism |
| OCAR_6017 | fructose-1 6-bisphosphatase class | 90 | Energy metabolism |
| OCAR_6702 | formate dehydrogenase alpha subunit | 91 | Energy metabolism |
| OCAR_4582 | oxoglutarate dehydrogenase | 93 | Energy metabolism |
| OCAR_5030 | nitrous-oxide reductase | 824 | Energy metabolism |
| OCAR_5440 | isocitrate dehydrogenase NADP-dependent | 72 | Energy metabolism |
| OCAR_4330 | acetate--CoA ligase | 77.8 | Energy metabolism |
| OCAR_4595 | ATP synthase F1 beta subunit | 675 | Energy metabolism |
| OCAR_6286 | pyruvate dehydrogenase | 100 | Energy metabolism |
| OCAR_7324 | NAD | 87.5 | Energy metabolism |
| OCAR_4581 | malate dehydrogenase | 82. 4 | Energy metabolism |
| OCAR_6963 | electron transfer flavoprotein-ubiquinone | 100 | Energy metabolism |
| OCAR_6702 | formate dehydrogenase alpha subunit | 100 | Energy metabolism |
| OCAR_7381 | D-beta-hydroxybutyrate dehydrogenas | 100 | Energy metabolism |
| OCAR_5125 | thioredoxin-disulfide reductase | 100 | Energy metabolism |
| OCAR_7138 | cytochrome c oxidase Cbb3-type subunit | 100 | Energy metabolism |
| OCAR_6769 | formate dehydrogenase-O major subunit | 100 | Energy metabolism |
| OCAR_7167 | L-lactate dehydrogenase | 100 | Energy metabolism |
| OCAR_6676 | formate dehydrogenase family access | 100 | Energy metabolism |
| OCAR_5321 | adenosylhomocysteinase | 62.5 | Energy metabolism |
| OCAR_4631 | mandelate racemase | 100 | Energy metabolism |
| OCAR_4992 | 6-phosphogluconate dehydrogenase | 80 | Energy metabolism |
| OCAR_6696 | L-carnitine dehydratase | 100 | Energy metabolism |
| OCAR_6560 | 2-hydroxy-3-oxopropionate reductase | 64 | Energy metabolism |
| OCAR_5019 | Dihydrolipoyllysine-residue succinyltransferase component | 100 | Energy metabolism |
| OCAR_5410 | fumarate hydratase class I anaerobic | 80 | Energy metabolism |
| OCAR_7510 | aryldialkylphosphatase | 100 | Fatty acid and phospholipid metabolism |
| OCAR_4106 | acetoacetyl-CoA reductase | 100 | Fatty acid and phospholipid metabolism |
| OCAR_5980 | acetyl-CoA carboxylase biotin carboxylase | 100 | Fatty acid and phospholipid metabolism |
| OCAR_4079 | acetyl-CoA carboxylase carboxyl transferase | 100 | Fatty acid and phospholipid metabolism |
| OCAR_6500 | beta-ketoacyl synthase | 60 | Fatty acid and phospholipid metabolism |
| OCAR_6842 | conserved hypothetical protein | 100 | Hypothetical proteins |
| OCAR_6577 | conserved hypothetical protein | 100 | Hypothetical proteins |
| OCAR_5964 | conserved hypothetical protein | 100 | Hypothetical proteins |
| OCAR_5605 | conserved hypothetical protein | 100 | Hypothetical proteins |
| OCAR_4731 | conserved hypothetical protein | 100 | Hypothetical proteins |
| OCAR_5300 | conserved hypothetical protein | 100 | Hypothetical proteins |
| OCAR_7083 | conserved hypothetical protein | 100 | Hypothetical proteins |
| OCAR_7620 | conserved hypothetical protein | 100 | Hypothetical proteins |
| OCAR_5413 | conserved hypothetical protein | 100 | Hypothetical proteins |
| OCAR_5858 | conserved hypothetical protein | 100 | Hypothetical proteins |
| OCAR_5868 | conserved hypothetical protein | 85.8 | Hypothetical proteins |
| OCAR_5293 | conserved hypothetical protein | 60 | Hypothetical proteins |
| OCAR_4417 | chaperone protein DnaK | 91. 7 | Protein fate |
| OCAR_4161 | heat shock protein Hsp20 | 100 | Protein fate |
| OCAR_5502 | chaperonin GroL | 82. 4 | Protein fate |
| OCAR_6288 | twin-arginine translocation pathway | 90 | Protein fate |
| OCAR_6535 | twin-arginine translocation pathway | 100 | Protein fate |
| OCAR_5907 | trigger factor | 100 | Protein fate |
| OCAR_5911 | ATP-dependent protease La | 100 | Protein fate |
| OCAR_4083 | PpiC-type peptidyl-prolyl cis-trans isomerase | 71.5 | Protein fate |
| OCAR_6040 | type I secretion membrane fusion protein | 100 | Protein fate |
| OCAR_7474 | twin-arginine translocation pathway | 100 | Protein fate |
| OCAR_4568 | heme exporter protein CcmC | 100 | Protein fate |
| OCAR_7492 | protein-L-isoaspartate | 100 | Protein fate |
| OCAR_5651 | thermosome alpha subunit | 100 | Protein fate |
| OCAR_5955 | ribosomal protein S2 | 100 | Protein synthesis |
| OCAR_6089 | valyl-tRNA synthetase | 100 | Protein synthesis |
| OCAR_5163 | L-seryl-tRNA selenium transferase | 100 | Protein synthesis |
| OCAR_7190 | ribosomal protein L25 Ctc-form | 100 | Protein synthesis |
| OCAR_4438 | phenylalanyl-tRNA synthetase betasubunit | 100 | Protein synthesis |
| OCAR_5674 | translation elongation factor G | 66.7 | Protein synthesis |
| OCAR_5947 | prolyl-tRNA synthetase | 100 | Protein synthesis |
| OCAR_5956 | translation elongation factor Ts | 100 | Protein synthesis |
| OCAR_6931 | dihydroorotase multifunctional component | 100 | Purines, pyrimidines, nucleosides, and nucleotides |
| OCAR_4020 | CadR | 83 | Regulatory functions |
| OCAR_7575 | transcriptional regulatory protein | 100 | Regulatory functions |
| OCAR_5653 | putative two-component sensor histidine kinase protein | 100 | Regulatory functions |
| OCAR_4994 | transcriptional regulator IclR-fam | 100 | Regulatory functions |
| OCAR_6413 | helix-turn-helix domain protein | 100 | Regulatory functions |
| OCAR_5985 | ribonuclease E (RNase E) | 80 | Regulatory functions |
| OCAR_5701 | DNA-directed RNA polymerase alpha | 61 | Transcription |
| OCAR_7055 | AAA_5 ATPase | 100 | Transcription |
| OCAR_6076 | ABC transporter substrate binding | 100 | Transport and binding proteins |
| OCAR_6079 | glutathione import ATP-binding protein | 100 | Transport and binding proteins |
| OCAR_6480 | extracellular ligand-binding receptor | 100 | Transport and binding proteins |
| OCAR_6707 | ATPase component of tungstate ABC transporter | 67.7 | Transport and binding proteins |
| OCAR_6394 | ABC transporter ATP-binding protein | 100 | Transport and binding proteins |
| OCAR_5649 | taurine transport system permease protein | 100 | Transport and binding proteins |
| OCAR_5477 | high-affinity branched-chain amino | 100 | Transport and binding proteins |
| OCAR_6658 | cation efflux system protein CzcA | 100 | Transport and binding proteins |
| OCAR_6967 | MFS permease | 100 | Transport and binding proteins |
| OCAR_4697 | H+-transporting two-sector ATPase | 100 | Transport and binding proteins |
| OCAR_5827 | Porin | 100 | Transport and binding proteins |
| OCAR_5119 | extracellular solute-binding protei | 88. 9 | Transport and binding proteins |
| OCAR_6418 | Y4BN | 51.6 | Transport and binding proteins |
| OCAR_7655 | Y4bN protein | 100 | Unclassified |
| OCAR_5218 | aminotransferase class IV | 75 | Unclassified |
| OCAR_7238 | peptidase M16 domain protein | 100 | Unknown function |
| OCAR_5612 | FHA domain containing protein | 100 | Unknown function |
| OCAR_6209 | PRC-barrel | 100 | Unknown function |
| OCAR_4313 | OsmC family protein | 100 | Unknown function |
| OCAR_4139 | oxidoreductase FAD/FMN-binding | 100 | Unknown function |
| OCAR_5938 | beta-lactamase domain protein | 100 | Unknown function |
| OCAR_5109 | FAD dependent oxidoreductase | 100 | Unknown function |
| OCAR_6433 | protein of unknown function | 100 | Unknown function |
| OCAR_4676 | tetratricopeptide TPR | 100 | Unknown function |
| OCAR_4947 | inositol monophosphatase family protein | 100 | Unknown function |
| OCAR_5308 | 2-oxo acid dehydrogenase acyltransferase | 100 | Unknown function |
| OCAR_5436 | UspA | 100 | Unknown function |
| gi\|47177065 | TraA [Oligotropha carboxidovorans] | 58 | Unknown function |
| OCAR_7060 | hypothetical protein | 100 |  |
| OCAR_5635 | hypothetical protein | 89 |  |
| gi\|47177082\|ref\|YP_015693.1\| | virB-11 like protein | 100 |  |
| gi\|47177046\|ref\|YP_015657.1\| | Methylase protein | 100 | Plasmid protein |
| gi\|47177036\|ref\|YP_015647.1\| | nitrogen-fixing protein | 100 | Plasmid protein |
| gi\|47177070\|ref\|YP_015681.1\| | Adhesin | 100 | Plasmid protein |
