# Supplementary material for: Proteome and Membrane Fatty Acid Analyses on Oligotropha carboxidovorans OM5 Grown under Chemolithoautotrophic and Heterotrophic Conditions
Source: PLoS One. 2011 Feb 28;6(2):e17111. doi: 10.1371/journal.pone.0017111 (PMC3046131; doi:10.1371/journal.pone.0017111)
Supplement: Table S5 — Proteins that significantly decreased in minimal medium with syngas compared to TSB (DOCX) [file pone.0017111.s006.docx]

Table S5. Proteins that significantly decreased in minimal medium with syngas compared to TSB

| *Locus tag* | *Protein name* | *% decrease* | *Main role category* |
| --- | --- | --- | --- |
| OCAR_4283 | 3-isopropylmalate dehydrogenase | 100 | Amino acid biosynthesis |
| OCAR_4938 | glutamate synthase [NADH] (nadh-gogat) | 100 | Amino acid biosynthesis |
| OCAR_4760 | argininosuccinate lyase | 100 | Amino acid biosynthesis |
| OCAR_5860 | serine hydroxymethyltransferase | 100 | Amino acid biosynthesis |
| OCAR_5892 | O-acetylhomoserine (thiol)-lyase (O-acetylhomoserinesulfhydrylase) | 100 | Amino acid biosynthesis |
| OCAR_6751 | 5-methyltetrahydropteroyltriglutamate--homocysteine S-methyltransfer | 100 | Amino acid biosynthesis |
| OCAR_4654 | acetylornithine aminotransferase | 100 | Amino acid biosynthesis |
| OCAR_4655 | ornithine carbamoyltransferase | 100 | Amino acid biosynthesis |
| OCAR_6061 | cysteine synthase A | 100 | Amino acid biosynthesis |
| OCAR_5901 | glutamine synthetase type I | 100 | Amino acid biosynthesis |
| OCAR_5292 | ketol-acid reductoisomerase | 94.8 | Amino acid biosynthesis |
| OCAR_6061 | cysteine synthase (O-acetylserine sulfhydrylase) | 100 | Amino acid biosynthesis |
| OCAR_6532 | molybdenum cofactor biosynthesis protein A | 100 | Biosynthesis of cofactors, prosthetic groups, and carriers |
| OCAR_7085 | 2-dehydropantoate 2-reductase | 100 | Biosynthesis of cofactors, prosthetic groups, and carriers |
| OCAR_5746 | gamma-glutamyltransferase | 100 | Biosynthesis of cofactors, prosthetic groups, and carriers |
| OCAR_4490 | 2-polyprenylphenol 6-hydroxylase | 100 | Biosynthesis of cofactors, prosthetic groups, and carriers |
| OCAR_6666 | 1-deoxy-D-xylulose-5-phosphate synthase | 100 | Biosynthesis of cofactors, prosthetic groups, and carriers |
| OCAR_4119 | 4-hydroxy-3-methylbut-2-en-1-yl diphosphate synthase | 100 | Biosynthesis of cofactors, prosthetic groups, and carriers |
| OCAR_5998 | cysteine desulfurase (Selenocysteine lyase) (Selenocysteine reductas | 100 | Biosynthesis of cofactors, prosthetic groups, and carriers |
| OCAR_5007 | FO synthase (7 8-didemethyl-8-hydroxy-5-deazariboflavinsynthase) | 100 | Biosynthesis of cofactors, prosthetic groups, and carriers |
| OCAR_5759 | pantoate--beta-alanine ligase | 100 | Biosynthesis of cofactors, prosthetic groups, and carriers |
| OCAR_5839 | pyridoxal phosphate biosynthetic protein PdxJ | 100 | Biosynthesis of cofactors, prosthetic groups, and carriers |
| OCAR_7258 | 4-hydroxybenzoate polyprenyl transferase | 100 | Biosynthesis of cofactors, prosthetic groups, and carriers |
| OCAR_5998 | cysteine desulfurase (Nitrogenase metalloclustersbiosynthesis protei | 100 | Biosynthesis of cofactors, prosthetic groups, and carriers |
| OCAR_7126 | uroporphyrinogen decarboxylase | 100 | Biosynthesis of cofactors, prosthetic groups, and carriers |
| OCAR_7134 | response regulator receiver | 100 | Cell envelope |
| OCAR_6489 | PepSY-associated TM helix domain protein | 100 | Cell envelope |
| OCAR_7191 | response regulator receiver | 100 | Cell envelope |
| OCAR_4293 | OmpA/MotB | 100 | Cell envelope |
| OCAR_6237 | outer-membrane immunogenic protein | 100 | Cell envelope |
| OCAR_7339 | immunogenic protein | 100 | Cell envelope |
| OCAR_6718 | OmpA domain protein transmembrane region-containing protein | 100 | Cell envelope |
| OCAR_4619 | glucose-1-phosphate cytidylyltransferase | 100 | Cell envelope |
| OCAR-4425 | membrane protein putative | 100 | Cell envelope |
| OCAR_6893 | histone deacetylase superfamily | 100 | Cell envelope |
| OCAR_5360 | putative membrane protein of unknown function | 100 | Cell envelope |
| OCAR_5056 | negative regulator of AmpC AmpD | 100 | Cell envelope |
| OCAR_7530 | inner membrane protein OxaA | 100 | Cell envelope |
| OCAR_4286 | membrane protein putative | 100 | Cell envelope |
| OCAR_6020 | lytic murein transglycosylase | 100 | Cell envelope |
| OCAR_6122 | udp-glucose 6-dehydrogenase (udp-glc dehydrogenase)(udp-glcdh) | 100 | Cell envelope |
| OCAR_5244 | UDP-N-acetylmuramate--alanine ligase | 100 | Cell envelope |
| OCAR_7082 | cellulose synthesis regulatory protein | 100 | Cell envelope |
| OCAR_4551 | glucose-1-phosphate thymidylyltransferase | 100 | Cell envelope |
| OCAR_6550 | formyl-CoA transferase | 100 | Cellular processes |
| OCAR_4891 | antibiotic biosynthesis monooxygenase | 100 | Cellular processes |
| OCAR_6551 | oxalyl-CoA decarboxylase | 100 | Cellular processes |
| OCAR_4107 | acetyl-CoA acetyltransferase (Acetoacetyl-CoA thiolase) | 100 | Cellular processes |
| OCAR_5733 | S-(hydroxymethyl)glutathione dehydrogenase/class III alcohol dehydrogenase | 100 | Cellular processes |
| OCAR_5736 | peroxiredoxin-6 | 100 | Cellular processes |
| OCAR_4902 | superoxide dismutase [Mn] (General stress protein 24)(GSP24) | 100 | Cellular processes |
| OCAR_7328 | hybrid peroxiredoxin hyPrx5 (Thioredoxin reductase) | 96 | Cellular processes |
| OCAR_4500 | Catalase | 98 | Cellular processes |
| OCAR_7754 | mercuric resistance operon regulatory protein | 100 | Cellular processes |
| OCAR_5717 | PEP phosphonomutase | 100 | Cellular processes |
| OCAR_5809 | methyl-accepting chemotaxis sensory transducer | 100 | Cellular processes |
| OCAR_4102 | hydroxyacylglutathione hydrolase | 100 | Cellular processes |
| OCAR_4080 | pollen allergen Poa pIX/Phl pVI C- | 100 | Cellular processes |
| OCAR_6708 | extracellular tungstate binding protein | 94.4 | Cellular processes |
| OCAR_4121 | Patatin | 100 | Cellular processes |
| OCAR_5970 | methyl-accepting chemotaxis sensory transducer | 100 | Cellular processes |
| OCAR_6744 | sulfoacetaldehyde acetyltransferase | 100 | Central intermediary metabolism |
| OCAR_7088 | formamidase (Formamide amidohydrolase) | 100 | Central intermediary metabolism |
| OCAR_5319 | methionine adenosyltransferase | 100 | Central intermediary metabolism |
| OCAR_6339 | polyphosphate kinase | 100 | Central intermediary metabolism |
| OCAR_4278 | carbonate dehydratase | 100 | Central intermediary metabolism |
| OCAR_6018 | haloacid dehalogenase type II | 100 | Central intermediary metabolism |
| OCAR_6562 | glyoxylate carboligase | 100 | Central intermediary metabolism |
| OCAR_4548 | sulfite reductase (NADPH) hemoprotein beta-component | 100 | Central intermediary metabolism |
| OCAR_5706 | zinc-binding dehydrogenase | 100 | Central intermediary metabolism |
| OCAR_6911 | short-chain dehydrogenase/reductase SDR | 100 | Central intermediary metabolism |
| OCAR_7443 | putative hydroxypyruvate reductase | 100 | Central intermediary metabolism |
| OCAR_7391 | NodQ bifunctional enzyme (Nodulation protein Q) | 100 | Central intermediary metabolism |
| OCAR_4042 | glyoxylate reductase (Glycolate reductase) | 100 | Central intermediary metabolism |
| OCAR_4597 | (Di)nucleoside polyphosphate hydrolase (Ap4Apyrophosphatase) | 100 | DNA metabolism |
| OCAR_4483 | double-strand break repair protein AddB | 100 | DNA metabolism |
| OCAR_6007 | DNA topoisomerase IV B subunit | 100 | DNA metabolism |
| OCAR_6210 | transcription-repair coupling factor | 100 | DNA metabolism |
| OCAR_6213 | ATP-dependent DNA helicase RecG | 100 | DNA metabolism |
| OCAR_4157 | ATP-dependent endonuclease family protein | 100 | DNA metabolism |
| OCAR_4844 | gene transfer agent | 100 | DNA metabolism |
| OCAR_4078 | tyrosine recombinase XerD | 100 | DNA metabolism |
| OCAR_5556 | DNA methylase N-4/N-6 domain protein | 100 | DNA metabolism |
| OCAR_5177 | sulfur oxidation protein | 100 | Energy metabolism |
| OCAR_5613 | Nitroreductase | 100 | Energy metabolism |
| OCAR_4684 | cytochrome c oxidase subunit II | 100 | Energy metabolism |
| OCAR_6723 | pkhd-type hydroxylase Blr3905 | 100 | Energy metabolism |
| OCAR_4289 | succinate dehydrogenase flavoprotein subunit | 100 | Energy metabolism |
| OCAR_7313 | fructose-bisphosphate aldolase class-I | 100 | Energy metabolism |
| OCAR_7324 | NAD | 100 | Energy metabolism |
| OCAR_5070 | Transaldolase | 100 | Energy metabolism |
| OCAR_6017 | fructose-1 6-bisphosphatase class II | 100 | Energy metabolism |
| OCAR_6702 | formate dehydrogenase alpha subunit | 100 | Energy metabolism |
| OCAR_7074 | methylisocitrate lyase | 100 | Energy metabolism |
| OCAR_4582 | oxoglutarate dehydrogenase (succinyl-transferring) E1 component | 100 | Energy metabolism |
| OCAR_5094 | NAD(P) transhydrogenase subunit alpha | 100 | Energy metabolism |
| OCAR_7310 | Transketolase | 100 | Energy metabolism |
| OCAR_5030 | nitrous-oxide reductase | 100 | Energy metabolism |
| OCAR_5440 | isocitrate dehydrogenase NADP-dependent | 100 | Energy metabolism |
| OCAR_6560 | 2-hydroxy-3-oxopropionate reductase | 100 | Energy metabolism |
| OCAR_7076 | 2-methylcitrate dehydratase 2 | 96.9 | Energy metabolism |
| OCAR_0332 | acetate--CoA ligase | 100 | Energy metabolism |
| OCAR_6291 | NADPH-dependent fmn reductase | 100 | Energy metabolism |
| OCAR_2539 | ribose 5-phosphate isomerase A | 100 | Energy metabolism |
| OCAR_6994 | carboxymethylenebutenolidase | 100 | Energy metabolism |
| OCAR_7311 | glyceraldehyde-3-phosphate dehydrogenase type I | 100 | Energy metabolism |
| OCAR_6834 | nitrate reductase alpha subunit | 100 | Energy metabolism |
| OCAR_7381 | D-beta-hydroxybutyrate dehydrogenase (bdh) | 100 | Energy metabolism |
| OCAR_4631 | mandelate racemase/muconate lactonizing enzyme C- domain protein | 100 | Energy metabolism |
| OCAR_2687 | formate dehydrogenase family accessory protein FdhD | 100 | Energy metabolism |
| OCAR_6778 | feruloyl-CoA synthetase | 100 | Energy metabolism |
| OCAR_4134 | photosynthetic apparatus regulatory protein RegA | 100 | Energy metabolism |
| OCAR-5159 | formate dehydrogenase alpha subunit | 100 | Energy metabolism |
| OCAR_6920 | glutaryl-CoA dehydrogenase (GCD) | 100 | Energy metabolism |
| OCAR_5116 | succinyl-coa:3-ketoacid-coenzyme a transferase subunit A | 100 | Energy metabolism |
| OCAR_5286 | NADH dehydrogenase i chain g | 100 | Energy metabolism |
| OCAR_6963 | electron transfer flavoprotein-ubiquinone oxidoreductase (ETF-QO) | 100 | Energy metabolism |
| OCAR_6285 | pyruvate dehydrogenase (acetyl-transferring) E1 component alpha subunit | 100 | Energy metabolism |
| OCAR_1981 | citrate (Si)-synthase | 91.9 | Energy metabolism |
| OCAR_6853 | probable arylformamidase (Kynurenine formamidase) (KF) | 100 | Energy metabolism |
| OCAR_4690 | cytochrome c oxidase Cbb3-type subunit III | 100 | Energy metabolism |
| OCAR_3180 | L-lactate dehydrogenase [cytochrome] | 100 | Energy metabolism |
| OCAR_5125 | thioredoxin-disulfide reductase | 100 | Energy metabolism |
| OCAR_6696 | L-carnitine dehydratase/bile acid-inducible protein F | 100 | Energy metabolism |
| OCAR_0574 | aconitate hydratase 1 | 100 | Energy metabolism |
| OCAR_5158 | formate dehydrogenase-O major subunit (Formatedehydrogenase-O subu | 100 | Energy metabolism |
| OCAR_5418 | dsba oxidoreductase | 100 | Energy metabolism |
| OCAR_4583 | dihydrolipoyllysine-residue succinyltransferase E2 component of ox | 100 | Energy metabolism |
| OCAR_5204 | malate dehydrogenase | 94 | Energy metabolism |
| OCAR_4106 | acetoacetyl-CoA reductase | 100 | Fatty acid and phospholipid metabolism |
| OCAR_4995 | carnitinyl-CoA dehydratase (Crotonobetainyl-CoAhydratase) | 100 | Fatty acid and phospholipid metabolism |
| OCAR_2510 | beta-ketoacyl synthase | 100 | Fatty acid and phospholipid metabolism |
| gi\|47177065 | acetyl-CoA carboxylase carboxyl transferase alpha subunit | 100 | Fatty acid and phospholipid metabolism |
| OCAR_5980 | acetyl-CoA carboxylase biotin carboxylase | 100 | Fatty acid and phospholipid metabolism |
| OCAR_1228 | acyl-coenzyme a dehydrogenase (acdh) | 100 | Fatty acid and phospholipid metabolism |
| OCAR_5032 | conserved hypothetical protein | 100 | Hypothetical proteins |
| OCAR_6365 | conserved hypothetical protein | 100 | Hypothetical proteins |
| OCAR_4715 | conserved hypothetical protein | 100 | Hypothetical proteins |
| OCAR_6142 | conserved hypothetical protein | 100 | Hypothetical proteins |
| OCAR_5856 | conserved hypothetical protein | 100 | Hypothetical proteins |
| OCAR_7734 | conserved hypothetical protein | 100 | Hypothetical proteins |
| OCAR_5294 | conserved hypothetical protein | 100 | Hypothetical proteins |
| OCAR_7484 | conserved hypothetical protein | 96.6 | Hypothetical proteins |
| OCAR_6497 | conserved hypothetical protein | 100 | Hypothetical proteins |
| OCAR_5151 | conserved hypothetical protein | 100 | Hypothetical proteins |
| OCAR_6192 | conserved hypothetical protein | 100 | Hypothetical proteins |
| OCAR_4962 | conserved hypothetical protein | 100 | Hypothetical proteins |
| OCAR_6497 | conserved hypothetical protein | 100 | Hypothetical proteins |
| OCAR_7020 | conserved hypothetical protein | 100 | Hypothetical proteins |
| OCAR_4623 | conserved hypothetical protein | 100 | Hypothetical proteins |
| OCAR_5348 | conserved hypothetical protein | 100 | Hypothetical proteins |
| OCAR_5881 | conserved hypothetical protein | 100 | Hypothetical proteins |
| OCAR_6710 | conserved hypothetical protein | 100 | Hypothetical proteins |
| OCAR_6927 | conserved hypothetical protein | 100 | Hypothetical proteins |
| OCAR_7083 | conserved hypothetical protein | 100 | Hypothetical proteins |
| OCAR_5881 | conserved hypothetical protein | 100 | Hypothetical proteins |
| OCAR_7199 | conserved hypothetical protein | 100 | Hypothetical proteins |
| OCAR_7489 | conserved hypothetical protein | 100 | Hypothetical proteins |
| OCAR_7708 | conserved hypothetical protein | 100 | Hypothetical proteins |
| OCAR_4541 | conserved hypothetical protein | 100 | Hypothetical proteins |
| OCAR_5711 | conserved hypothetical protein | 100 | Hypothetical proteins |
| OCAR_5915 | conserved hypothetical protein | 100 | Hypothetical proteins |
| OCAR_4936 | conserved hypothetical protein | 100 | Hypothetical proteins |
| OCAR_4630 | conserved hypothetical protein | 100 | Hypothetical proteins |
| OCAR_5605 | conserved hypothetical protein | 100 | Hypothetical proteins |
| OCAR_6151 | phage integrase | 100 | Mobile and extrachromosomal element functions |
| OCAR_4974 | integrase catalytic region | 100 | Mobile and extrachromosomal element functions |
| OCAR_4083 | PpiC-type peptidyl-prolyl cis-trans isomerase | 100 | Protein fate |
| OCAR_4082 | preprotein translocase SecA subunit | 100 | Protein fate |
| OCAR_5047 | peptidyl-prolyl cis-trans isomerase cyclophilin-type | 100 | Protein fate |
| OCAR_3285 | chaperonin GroL | 100 | Protein fate |
| OCAR_6535 | twin-arginine translocation pathway signal | 100 | Protein fate |
| OCAR_6649 | twin-arginine translocation pathway signal | 98.8 | Protein fate |
| OCAR_4896 | twin-arginine translocation pathway signal | 100 | Protein fate |
| OCAR_4057 | peptidase M48 Ste24p | 100 | Protein fate |
| OCAR_4429 | twin-arginine translocation pathway signal | 100 | Protein fate |
| OCAR_7271 | chaperonin GroS | 100 | Protein fate |
| OCAR_5732 | twin-arginine translocation pathway signal | 100 | Protein fate |
| OCAR_5907 | trigger factor | 100 | Protein fate |
| OCAR_5908 | Clp protease | 100 | Protein fate |
| OCAR_4130 | penicillin-binding protein 1A (PBP-1a) (PBP1a) | 100 | Protein fate |
| OCAR_5037 | peptidase U32 | 100 | Protein fate |
| OCAR_7492 | protein-L-isoaspartate | 100 | Protein fate |
| OCAR_7450 | secretion protein HlyD | 100 | Protein fate |
| OCAR_5651 | thermosome alpha subunit | 100 | Protein fate |
| gi\|47177070 | carboxy--processing protease (C-terminal-processing protease) | 100 | Protein fate |
| OCAR_6040 | type I secretion membrane fusion protein HlyD | 100 | Protein fate |
| OCAR_6256 | ATP-dependent protease subunit | 100 | Protein fate |
| OCAR_7450 | secretion protein HlyD | 100 | Protein fate |
| OCAR_4568 | heme exporter protein CcmC | 100 | Protein fate |
| OCAR_6949 | glycyl-tRNA synthetase beta subunit | 100 | Protein synthesis |
| OCAR_5955 | ribosomal protein S2 | 100 | Protein synthesis |
| OCAR_6374 | ribosomal protein L9 | 100 | Protein synthesis |
| OCAR_7469 | glutamyl-tRNA(Gln) amidotransferase subunit A (Glu-ADTsubunit A) | 100 | Protein synthesis |
| OCAR_1683 | translation elongation factor G | 100 | Protein synthesis |
| OCAR_4370 | tRNA uridine 5-carboxymethylaminomethyl modification enzyme GidA | 100 | Protein synthesis |
| OCAR_4508 | translation initiation factor IF-2 | 100 | Protein synthesis |
| OCAR_1214 | tRNA (5-methylaminomethyl-2-thiouridylate)-methyltransferase | 100 | Protein synthesis |
| OCAR_1169 | L-seryl-tRNA selenium transferase | 100 | Protein synthesis |
| OCAR_2034 | translation elongation factor P | 100 | Protein synthesis |
| OCAR_6125 | methionyl-tRNA synthetase | 93.8 | Protein synthesis |
| OCAR_7190 | ribosomal protein L25 Ctc-form | 100 | Protein synthesis |
| OCAR_0524 | tRNA-I(6)A37 thiotransferase enzyme MiaB | 100 | Protein synthesis |
| OCAR_5947 | prolyl-tRNA synthetase | 100 | Protein synthesis |
| OCAR_6334 | aspartyl-tRNA synthetase | 100 | Protein synthesis |
| OCAR_6931 | dihydroorotase multifunctional complex type | 100 | Purines, pyrimidines, nucleosides, and nucleotides |
| OCAR_6346 | nucleoside diphosphate kinase (NDK) (NDP kinase) | 100 | Purines, pyrimidines, nucleosides, and nucleotides |
| OCAR_6754 | ribonucleoside-diphosphate reductase alpha subunit | 100 | Purines, pyrimidines, nucleosides, and nucleotides |
| OCAR_6755 | ribonucleoside-diphosphate reductase beta subunit | 100 | Purines, pyrimidines, nucleosides, and nucleotides |
| OCAR_3146 | transcriptional regulatory protein FixJ | 100 | Regulatory functions |
| OCAR_4424 | transcriptional regulator AraC family | 100 | Regulatory functions |
| OCAR_6413 | helix-turn-helix domain protein | 100 | Regulatory functions |
| OCAR_4362 | diguanylate cyclase | 100 | Regulatory functions |
| OCAR_6443 | type IV secretory pathway VirD2 components | 100 | Regulatory functions |
| OCAR_4739 | membrane protein involved in aromatic hydrocarbon degradation | 100 | Regulatory functions |
| OCAR_7201 | two-component hybrid sensor and regulator | 100 | Regulatory functions |
| OCAR_5653 | putative two-component sensor histidine kinase protein | 100 | Regulatory functions |
| OCAR_6745 | transcriptional regulator IclR-family | 100 | Regulatory functions |
| OCAR_4020 | CadR | 100 | Regulatory functions |
| OCAR_7493 | putative sensor histidine kinase | 100 | Regulatory functions |
| OCAR_7575 | transcriptional regulatory protein ChvI | 100 | Regulatory functions |
| OCAR_5130 | transcription elongation factor GreA | 100 | Transcription |
| OCAR_5663 | DNA-directed RNA polymerase beta subunit | 100 | Transcription |
| OCAR_4503 | polyribonucleotide nucleotidyltransferase | 100 | Transcription |
| OCAR_1666 | transcription termination/antitermination factor NusG | 100 | Transcription |
| OCAR_5985 | ribonuclease E (RNase E) | 100 | Transcription |
| OCAR_5701 | DNA-directed RNA polymerase alpha subunit | 100 | Transcription |
| OCAR_5328 | lipid A export ATP-binding/permease protein MsbA | 100 | Transport and binding proteins |
| OCAR_7360 | binding-protein-dependent transport systems inner membrane component | 100 | Transport and binding proteins |
| OCAR_6187 | cation diffusion facilitator family transporter | 100 | Transport and binding proteins |
| OCAR_4635 | general L-amino acid-binding periplasmic protein AapJ | 100 | Transport and binding proteins |
| OCAR_5475 | Leu/Ile/Val-binding protein (LIV-BP) | 100 | Transport and binding proteins |
| OCAR_5119 | extracellular solute-binding protein family 1 | 100 | Transport and binding proteins |
| OCAR_5867 | V-type H(+)-translocating pyrophosphatase | 100 | Transport and binding proteins |
| OCAR_7637 | lead cadmium zinc and mercury-transporting ATPase | 100 | Transport and binding proteins |
| OCAR_5827 | Porin | 100 | Transport and binding proteins |
| OCAR_6880 | ABC-type nitrate/sulfonate/bicarbonate transport systems periplasmic | 100 | Transport and binding proteins |
| OCAR_7386 | thiosulfate-binding protein | 100 | Transport and binding proteins |
| OCAR_4434 | extracellular solute-binding protein family 5 | 100 | Transport and binding proteins |
| OCAR_6480 | extracellular ligand-binding receptor | 100 | Transport and binding proteins |
| OCAR_4126 | ABC transporter substrate-binding protein aliphatic sulphonates | 100 | Transport and binding proteins |
| OCAR_5119 | extracellular solute-binding protein family 1 | 100 | Transport and binding proteins |
| OCAR_5297 | C4-dicarboxylate-binding periplasmic protein | 97.7 | Transport and binding proteins |
| OCAR_6537 | periplasmic mannitol-binding protein | 100 | Transport and binding proteins |
| OCAR_6394 | ABC transporter ATP-binding protein | 100 | Transport and binding proteins |
| OCAR_7512 | ABC transporter substrate binding protein [dipeptide] | 100 | Transport and binding proteins |
| OCAR_7040 | Bacterioferritin | 100 | Transport and binding proteins |
| OCAR_4968 | putative flavin-nucleotide-binding protein | 100 | Transport and binding proteins |
| OCAR_6707 | ATPase component of tungstate ABC transporter | 100 | Transport and binding proteins |
| OCAR_7055 | AAA_5 ATPase | 100 | Transport and binding proteins |
| OCAR_4667 | extracellular solute-binding protein family 3 | 100 | Transport and binding proteins |
| OCAR_5029 | periplasmic copper-binding | 100 | Transport and binding proteins |
| OCAR_7428 | glutathione import ATP-binding protein GsiA | 100 | Transport and binding proteins |
| OCAR_1206 | monovalent cation/proton antiporter | 100 | Transport and binding proteins |
| OCAR_6538 | MRP ATP/GTP-binding protein | 100 | Transport and binding proteins |
| OCAR_4633 | ferrous iron transport protein B | 100 | Transport and binding proteins |
| OCAR_7431 | bacterial extracellular solute-binding proteins family 5 | 100 | Transport and binding proteins |
| OCAR_4697 | H+-transporting two-sector ATPase B/Bp subunit | 100 | Transport and binding proteins |
| OCAR_7499 | abc-type transporter periplasmic component: haat family | 100 | Transport and binding proteins |
| OCAR_5305 | MFS permease | 100 | Transport and binding proteins |
| OCAR_6658 | cation efflux system protein CzcA | 100 | Transport and binding proteins |
| OCAR_6181 | CumA | 100 | Unclassified |
| OCAR_7655 | Y4bN protein | 100 | Unclassified |
| OCAR_6418 | Y4BN | 100 | Unclassified |
| OCAR_5612 | FHA domain containing protein | 100 | Unknown function |
| OCAR_7185 | protein of unknown function | 100 | Unknown function |
| OCAR_4840 | glycoside hydrolase family 17 | 100 | Unknown function |
| OCAR_4066 | bordetella uptake gene (bug) product superfamily | 100 | Unknown function |
| OCAR_5436 | UspA | 100 | Unknown function |
| OCAR_4139 | oxidoreductase FAD/FMN-binding | 100 | Unknown function |
| OCAR_6988 | LemA | 100 | Unknown function |
| OCAR_4841 | putative homologue of Rhodobacter capsulatus gene transfer agent | 100 | Unknown function |
| OCAR_6209 | PRC-barrel | 100 | Unknown function |
| OCAR_5709 | AAA ATPase central region | 100 | Unknown function |
| OCAr_5218 | aminotransferase class IV | 100 | Unknown function |
| OCAR_7238 | peptidase M16 domain protein | 100 | Unknown function |
| gi\|47177036 | conserved hypothetical protein putative | 100 | Unknown function |
| OCAR_5938 | beta-lactamase domain protein | 100 | Unknown function |
| OCAR_5308 | 2-oxo acid dehydrogenase acyltransferase catalytic domain protein | 100 | Unknown function |
| OCAR_7039 | protein of unknown function putative | 100 | Unknown function |
| OCAR_4676 | tetratricopeptide TPR_2 | 100 | Unknown function |
| OCAR_4947 | inositol monophosphatase family protein | 100 | Unknown function |
| OCAR_5087 | ATP/GTP-binding site motif A | 100 | Unknown function |
| OCAR_6647 | peptidase C15 pyroglutamyl peptidase I | 100 | Unknown function |
| OCAR_4856 | OsmC family protein | 100 | Unknown function |
| OCAR_5436 | UspA | 100 | Unknown function |
| OCAR_5109 | FAD dependent oxidoreductase | 100 | Unknown function |
| OCAR_4038 | hypothetical protein | 100 |  |
| OCAR_7060 | hypothetical protein | 100 |  |
| OCAR_4448 | [Ni/Fe] hydrogenase maturation protein | 100 |  |
| gi\|47177028 | TraA [Oligotropha carboxidovorans] | 100 |  |
| OCAR_4155 | hypothetical protein | 100 |  |
| OCAR_7704 | VirB11-like protein | 100 |  |
| OCAR_5080 | nitrogen-fixing protein | 100 |  |
| OCAR_5910 | hypothetical protein | 100 |  |
| OCAR_4081 | hypothetical protein | 100 |  |
| OCAR_5635 | hypothetical protein | 100 |  |
| OCAR_7108 | adhesin | 100 |  |
