# Supplementary material for: Proteome and Membrane Fatty Acid Analyses on Oligotropha carboxidovorans OM5 Grown under Chemolithoautotrophic and Heterotrophic Conditions
Source: PLoS One. 2011 Feb 28;6(2):e17111. doi: 10.1371/journal.pone.0017111 (PMC3046131; doi:10.1371/journal.pone.0017111)
Supplement: Table S6 — Proteins that significantly increased in minimal medium with syngas compared to TSB. (DOCX) [file pone.0017111.s007.docx]

Table S6. Proteins that significantly increased in minimal medium with syngas compared to TSB

| *Locus tag* | *Protein name* | *%increase* | *Main role category* |
| --- | --- | --- | --- |
| OCAR_4480 | FolC bifunctional protein | 960 | Biosynthesis of cofactors, prosthetic groups, and carriers |
| OCAR_5999 | FeS assembly protein SufB | 930 | Biosynthesis of cofactors, prosthetic groups, and carriers |
| OCAR_4023 | hypothetical protein | 100 | Cell envelope |
| OCAR_5236 | peptidoglycan glycosyltransferase | 838 | Cell envelope |
| OCAR_5491 | dolichyl-phosphate beta-D-mannosyltransferase | 97 | Cell envelope |
| OCAR_4348 | tetraacyldisaccharide 4prime-kinase | 100 | Cell envelope |
| OCAR_7731 | membrane protein putative | 85 | Cell envelope |
| OCAR_7602 | conjugal transfer protein TrbL | 87.8 | Cell envelope |
| OCAR_7736 | putative exported protein of unknown function | 72 | Cell envelope |
| OCAR_6064 | multidrug resistance protein MdtB (Multidrug transporter mdtB) | 95 | Cellular processes |
| OCAR_0131 | protein-P-II uridylyltransferase | 100 | Central intermediary metabolism |
| OCAR_5483 | 3-oxoadipate enol-lactonase | 100 | Energy metabolism |
| OCAR_6675 | Fe-S oxidoreductase | 98 | Energy metabolism |
| OCAR_6776 | glutaconate CoA-transferase subunit A | 95.6 | Energy metabolism |
| OCAR_5771 | glycerophosphoryl diester phosphodiesterase | 95.8 | Fatty acid and phospholipid metabolism |
| OCAR_4039 | conserved hypothetical protein | 93 | Hypothetical proteins |
| OCAR_4927 | conserved hypothetical protein | 100 | Hypothetical proteins |
| OCAR_5689 | 50S ribosomal protein L5 | 100 | Mobile and extrachromosomal element functions |
| OCAR_4286 | membrane protein putative | 98 | Protein fate |
| OCAR_4057 | peptidase M48 Ste24p | 95 | Protein fate |
| OCAR_7426 | cytosol aminopeptidase (Leucine aminopeptidase) (LAP) | 100 | Protein fate |
| OCAR_5401 | multi-sensor hybrid histidine kinase | 100 | Protein synthesis |
| OCAR_1684 | translation elongation factor Tu | 69 | Protein synthesis |
| OCAR_6104 | methyltransferase type 11 | 94 | Protein synthesis |
| OCAR_4110 | diguanylate cyclase/phosphodiesterase | 92 | Regulatory functions |
| OCAR_6097 | nitrogen regulation protein NtrY | 83.8 | Regulatory functions |
| OCAR_4064 | membrane protein Mlr2225 | 100 | Regulatory functions |
| OCAR_4705 | C4-dicarboxylate transport transcriptional regulatory protein | 100 | Transport and binding proteins |
| OCAR_5649 | taurine transport system permease protein TauC | 92.8 | Transport and binding proteins |
| OCAR_4899 | branched-chain amino acid ABC transporter permease protein | 100 | Transport and binding proteins |
| OCAR_4391 | MltA | 84.7 | Unclassified |
| OCAR_5854 | RDD | 100 | Unknown function |
| OCAR_4066 | bordetella uptake gene (bug) product superfamily | 77.7 | Unknown function |
| OCAR_4549 | hypothetical protein | 100 | Unknown function |
| OCAR_5614 | hypothetical protein | 91 | Unknown function |
