# Supplementary material for: Proteome and Membrane Fatty Acid Analyses on Oligotropha carboxidovorans OM5 Grown under Chemolithoautotrophic and Heterotrophic Conditions
Source: PLoS One. 2011 Feb 28;6(2):e17111. doi: 10.1371/journal.pone.0017111 (PMC3046131; doi:10.1371/journal.pone.0017111)
Supplement: Table S7 — Proteins with significantly increased (P≤0.05) quantities in the presence of syngas compared to TSB and/or AC. (DOCX) [file pone.0017111.s008.docx]

Table S7. Proteins with significantly increased **(**P≤0.05**)** quantities in the presence of syngas compared to TSB and/or AC. The complete list of significantly altered proteins in TSB, AC, and SYN is provided in Tables S1, S2, S3, S4, S5, and S6

| **Product name** | **GI** | **COGs** | **%increase*** | |  |  |
| --- | --- | --- | --- | --- | --- | --- |
|  |  |  | **relative to TSB** | **relative to acetate** |  |  |
| **Biosynthesis/degradation** | | | | |  |  |
| Fe-S oxidoreductase | 209885794 | COG1032C | 98.1 | 100 |  |  |
| Glycerophosphoryl diester phosphodiesterase | 209884905 | COG0584C | 95.7 | 100 |  |  |
| Homospermidine synthase (HSS) | 209884057 | COG5310Q | 100 | 100 |  |  |
| 3-oxoadipate enol-lactonase | 209884623 | COG0596R | 100 | 100 |  |  |
| succinyl-CoA synthetase beta chain | 209885674 | COG0045C | NDC† | 83.0 |  |  |
| methylmalonate-semialdehyde dehydrogenase | 209886615 | COG1012C | NDC | 70.9 |  |  |
| dolichyl-phosphate beta-D-mannosyltransferase | 209884631 | COG0463M,COG2246S | 97.1 | 100 |  |  |
| peptidase M48 Ste24p | 209883216 | COG4784R | NDC | 100 |  |  |
| riboflavin biosynthesis protein RibF | 209886344 | COG0196H | 100 | 100 |  |  |
| FolC bifunctional protein | 209883633 | COG0285H | 96.2 | 100 |  |  |
| cytosol aminopeptidase (Leucine aminopeptidase) | 209885473 | COG0260E | 100 | 100 |  |  |
| Aryldialkylphosphatase | 209886621 | COG1228Q | 100 | 100 |  |  |
| sulfite reductase | 209883700 | COG0369P | NDC | 93.8 |  |  |
| ATP phosphoribosyltransferase | 209885425 | COG0547E | 50 | 92.9 |  |  |
| NADH dehydrogenase (quinone) g subunit | 209884426 | COG3383R | 100 | 100 |  |  |
| **Cell wall metabolism** | | | | |  |  |
| **Membrane-bound lytic murein transglycosylase A** (MltA) | 209883545 | COG2821M | 84.6 | 83.3 |  |  |
| diaminopimelate epimerase | 209883420 | COG0253E | 100 | 100 |  |  |
| peptidoglycan glycosyltransferase | 209885543 | COG0768M | 83.7 | 100 |  |  |
| **Gene regulation** | | | | |  |  |
| diguanylate cyclase/phosphodiesterase | 209884905 | COG0584C | NDC | 93.16 |  |  |
| multi-sensor hybrid histidine kinase | 209886204 | COG0642T,COG2204T | 100 | 100 |  |  |
| nitrogen regulation protein NtrY | 209885220 | COG5000T | 83.8 | 53.7 |  |  |
| C4-dicarboxylate transport transcriptional regulatory protein | 209883855 | COG4566T | 100 | 100 |  |  |
| **Conjugation** | | | | |  |  |
| conjugal transfer protein TrbL | 209885543 | COG2948U | 87.7 | 65.9 |  |  |
| **Transport** | | | | |  |  |
| multidrug resistance protein | 209885189 | COG0841V | 95.1 | 56.9 |  |  |
| cation efflux system protein CzcA | 209886831 | COG3696P | NDC | 95.6 |  |  |
| ABC transporter permease protein | 209883528 | COG0600P | 93.0 | 93.3 |  |  |
| taurine transport system permease protein TauC | 209884786 | COG0600P | 92.5 | 100 |  |  |
| **Others** | | | | |  |  |
| DNA polymerase I (POL I) | 209886676 | COG0258L,COG0749L | 100 | 100 |  |  |
| FeS assembly protein SufB | 209885127 | COG0719O | 93.1 | 95.7 |  |  |
| Thioesterase superfamily | 209886192 | COG1607I | 100 | 100 | |  |
| Ppx/GppA phosphatase | 209885459 | COG0248FP | 100 | 100 | |  |
| bordetella uptake gene (bug) product superfamily | 209883225 | COG3181S | 77.7 | 90 | | |
| DNA-directed RNA polymerase betap subunit | 209884799 | COG0085K | NDC | 93.2 | | |
| tetraacyldissaccharide 4-kinase | 209883502 | COG2835S | 100 | 100 | | |
| methyltransferase type 11 | 209885227 | COG0500QR | 94.9 | 77.9 | | |

*Percent increase was calculated as the ratio of the increase in ΣXcorrs (syngas compared to acetate or TSB) relative to the ΣXcorrs in acetate or TSB for each protein, expressed as a percentage.

†NDC. No detectable change.
